# Comparative genomics reveals structural and functional features specific to the genome of a foodborne *Escherichia coli* O157:H7

**DOI:** 10.1186/s12864-019-5568-6

**Published:** 2019-03-08

**Authors:** Vijay K. Sharma, Suryatej Akavaram, Robert G. Schaut, Darrell O. Bayles

**Affiliations:** 1 Food Safety and Enteric Pathogens Research Unit, USDA, ARS, National Animal Disease Center, 1920 Dayton Avenue, P.O. Box 70, Ames, IA 50010 USA; 2 Infectious Bacterial Diseases Research Unit, National Animal Disease Center, ARS-USDA, Ames, Iowa USA; 3 Oak Ridge Institute for Science and Education (ORISE), ARS Research Participation Program, MS 36, P.O. Box 117, Oak Ridge, TN 37831 USA

**Keywords:** O157, Genomics, Bacteriophages, Mobile elements, Genomic islands, Stress response

## Abstract

**Background:**

*Escherichia coli* O157:H7 (O157) has been linked to numerous foodborne disease outbreaks. The ability to rapidly sequence and analyze genomes is important for understanding epidemiology, virulence, survival, and evolution of outbreak strains. In the current study, we performed comparative genomics to determine structural and functional features of the genome of a foodborne O157 isolate NADC 6564 and infer its evolutionary relationship to other O157 strains.

**Results:**

The chromosome of NADC 6564 contained 5466 kb compared to reference strains Sakai (5498 kb) and EDL933 (5547 kb) and shared 41 of its 43 Linear Conserved Blocks (LCB) with the reference strains. However, 18 of 41 LCB had inverse orientation in NADC 6564 compared to the reference strains. NADC 6564 shared 18 of 19 bacteriophages with reference strains except that the chromosomal positioning of some of the phages differed among these strains. The additional phage (P19) of NADC 6564 was located on a 39-kb insertion element (IE) encoding several hypothetical proteins, an integrase, transposases, transcriptional regulators, an adhesin, and a phosphoethanolamine transferase (PEA). The complete homologs of the 39-kb IE were found in *E. coli* PCN061 of porcine origin. The IE-encoded PEA showed low homology (32–33%) to four other PEA in NADC 6564 and PEA linked to mobilizable colistin resistance in *E. coli* but was highly homologous (95%) to a PEA of uropathogenic, avian pathogenic, and enteroaggregative *E. coli*. NADC 6564 showed slightly higher minimum inhibitory concentration of colistin compared to the reference strains. The 39-kb IE also contained *dndBCDE* and *dptFGH* operons encoding DNA S-modification and a restriction pathway, linked to oxidative stress tolerance and self-defense against foreign DNA, respectively. Evolutionary tree analysis grouped NADC 6564 with lineage I O157 strains.

**Conclusions:**

These results indicated that differential phage counts and different chromosomal positioning of many bacteriophages and genomic islands might have resulted in recombination events causing altered chromosomal organization in NADC 6564. Evolutionary analysis grouped NADC 6564 with lineage I strains and suggested its earlier divergence from these strains. The ability to perform S-DNA modification might affect tolerance of NADC 6564 to various stressors.

**Electronic supplementary material:**

The online version of this article (10.1186/s12864-019-5568-6) contains supplementary material, which is available to authorized users.

## Background

Enterohemorrhagic *Escherichia coli* O157:H7 (O157) is a zoonotic human pathogen, transmitted through the consumption of contaminated foods, such as beef and dairy products, ready-to-eat salad greens, vegetables, and fruits [1–5]. O157 infections are the predominant cause of hemorrhagic uremic syndrome and kidney failure, especially in children and elderly [6, 7]. Cattle are the primary reservoir of O157 and are asymptomatic carriers of these bacteria in their gastrointestinal tract [8]. Carrier cattle shed O157 in their feces, which is a major risk factor in the contamination of meats produced from these animals [4, 9–11]. Since the first reported disease outbreak linked to O157 in the early 1980s [12], numerous outbreaks implicating O157 have occurred in the USA [13].

A variety of approaches, such as pulse-field gel electrophoresis [14, 15], multilocus genotyping [16–19], PCR- or microarray-based DNA fingerprinting [20–22], single nucleotide polymorphism (SNP) analysis [23, 24], and whole genome sequencing have been used to characterize outbreak strains and to understand their epidemiology, virulence, and evolutionary relatedness [25–28]. Some of these approaches have allowed grouping of O157 isolates into lineages I, I/II, and II. The isolates in lineages I and I/II are predominantly recovered from infected humans and those in lineage II are predominantly cattle-associated [21, 29]. Several lineage I isolates that have been linked to large disease outbreaks possess genetic alterations that cause increased expression of virulence genes, thus enhancing morbidity and the likelihood of serious disease in infected individuals [27, 30, 31]. In addition, genetic variability caused by single nucleotide polymorphisms or insertion and deletions in certain loci are known to enhance expression of genes linked to biofilm formation among O157 isolates [32–36]. It has been postulated that O157 isolates exist as heterogeneous populations capable of expressing genes for colonizing biotic surfaces, such as gastrointestinal tracts of carrier animals and incidental human host as well as surfaces of leafy vegetables, and abiotic surfaces through the formation of biofilms [29, 31–34, 37–44].

Whole genome sequencing of several O157 isolates has revealed that horizontally-acquired DNA, called O islands, in the core of the *E. coli* genome has contributed to the emergence of pathogenic O157 strains from a non-pathogenic ancestral *E. coli* [25, 45, 46]. Many of these O islands represent mobile elements, such as bacteriophages or insertion elements, encoding functions related to virulence [25, 47]. Prominent among these are the locus of enterocyte effacement (LEE) and genes encoding for Shiga toxins [25, 48]. While LEE promotes intimate adherence of O157 to intestinal epithelial cells [49, 50], Shiga toxins damage microvasculature of intestinal mucosa and kidneys to produce hemorrhagic colitis and hemorrhagic uremic syndrome, respectively [51]. According to a recent phylogenetic analysis, O157 strains once shared a common ancestor with enteropathogenic *E. coli* (EPEC) O55:H7 and then diverged from EPEC [46]. This divergence is characterized by the acquisition of plasmid pO157, bacteriophages encoding Shiga toxins 1 and 2, a novel O antigen-encoding gene cluster, and by loss of functions, such as the ability to ferment sorbitol and express beta-glucuronidase [52, 53]. A subsequent report estimated that the average mutation rate is about 50% faster in the O157 lineage compared to the O55:H7 lineage, suggesting a more recent divergence of these two lineages as opposed to an earlier divergence time point [46].

Besides acquiring virulence genes through horizontal gene transfer, bacterial pathogens may also acquire genomic islands encoding genes for survival under multiple stresses. The *dnd* operon, originally identified in *Streptomyces lividans* 66, encodes enzymes for sequence-specific, post-replication swapping of non-bridging oxygen with a sulfur in the DNA backbone [54, 55]. This DNA S-modification has been shown to enable bacterial growth under a variety of stressful conditions, such as extreme temperature, pH, UV, X-ray, salinity, pressure and heavy metals, by protecting genomic DNA and proteins from intracellular oxidative damage [56]. Not all, but many bacterial species harboring *dnd* genes also carry an upstream, three-gene operon (*dptFGH*) encoding a DNA S-modification-dependent restriction system for restricting heterologous DNA and protecting the host S-modified DNA [57]. The genes in the *dnd* and *dpt* operons, which have been identified in phylogenetically diverse bacterial species, including both non-pathogenic and pathogenic *E. coli* [58], exhibit a high degree of synteny. The presence of these operons on genomic islands or mobile elements indicates that these genes spread from an ancestral species to other bacterial species during the course of evolution [59].

Similarly, acquisition of antibiotic resistance genes via mobile DNA elements enhances bacterial survival in the presence of antibiotics. Colistin has been a last-resort treatment option for multidrug-resistant gram-negative pathogens, including members of Enterobacteriaceae; however, the emergence of colistin resistance had rendered this antibiotic ineffective. One of the mechanisms enhancing resistance to colistin involves phosphoethanolamine substitution in the outer membrane lipid A that reduces the ability of colistin to enter and kill bacterial cells [60]. The enzymes phosphoethanolamine transferases (PEA) constitute a family of related proteins that are responsible for lipid A modification. Some of these PEA have evolved to confer a high level of mobilizable colisitin resistance (MCR), which is encoded by the *mcr* genes present on mobile elements that are capable of transmission to other gram-negative bacterial species [60, 61]. According to a recent report, O157 carries a chromosomal PEA gene *pmrC* and several other PEA genes mediating lipid A modification that contribute to a slight increase in the minimum inhibitory concentration of colistin and other cationic antibiotics [62]. It has been suggested that PEA-mediated lipid A modification could confer survival advantage to O157 in certain environmental niches [62].

The aim of the current study was to perform comparative genomics of a Shiga toxin-producing (STEC) *E. coli* O157:H7 (O157) strain (str.) NADC 6564 with other STEC O157 and non-O157 STEC strains isolated in the past from different food outbreaks or from cattle. The strain NADC 6564 is a Congo red-negative isolate of a 1986 foodborne STEC O157:H7 str. 86–24 [28], which has been used extensively in live animal models (mice and cattle) as well in tissue culture assays to study O157 virulence and ability to colonize carrier animals intestines and produce biofilms on abiotic matrices [63–71]. We have recently published a complete genome sequence of NADC 6564 and its Congo red-positive variant (str. NADC 6565) wherein we provided basic information about these two genomes and highlighted genetic alterations conferring Congo red-positive and biofilm-producing ability on NADC 6565 [28]. The major emphasis of the current study was to use comparative genomic approaches to identify genetic features unique to str. NADC 6564 and to infer if any of these features could have direct or indirect impact on virulence, ability to colonize the host animal intestine, and survival in the external environment compared to the other STEC O157 strains linked to various food outbreaks in the past.

## Results

### General features of strain NADC 6564 chromosome

**Fig. 1:**
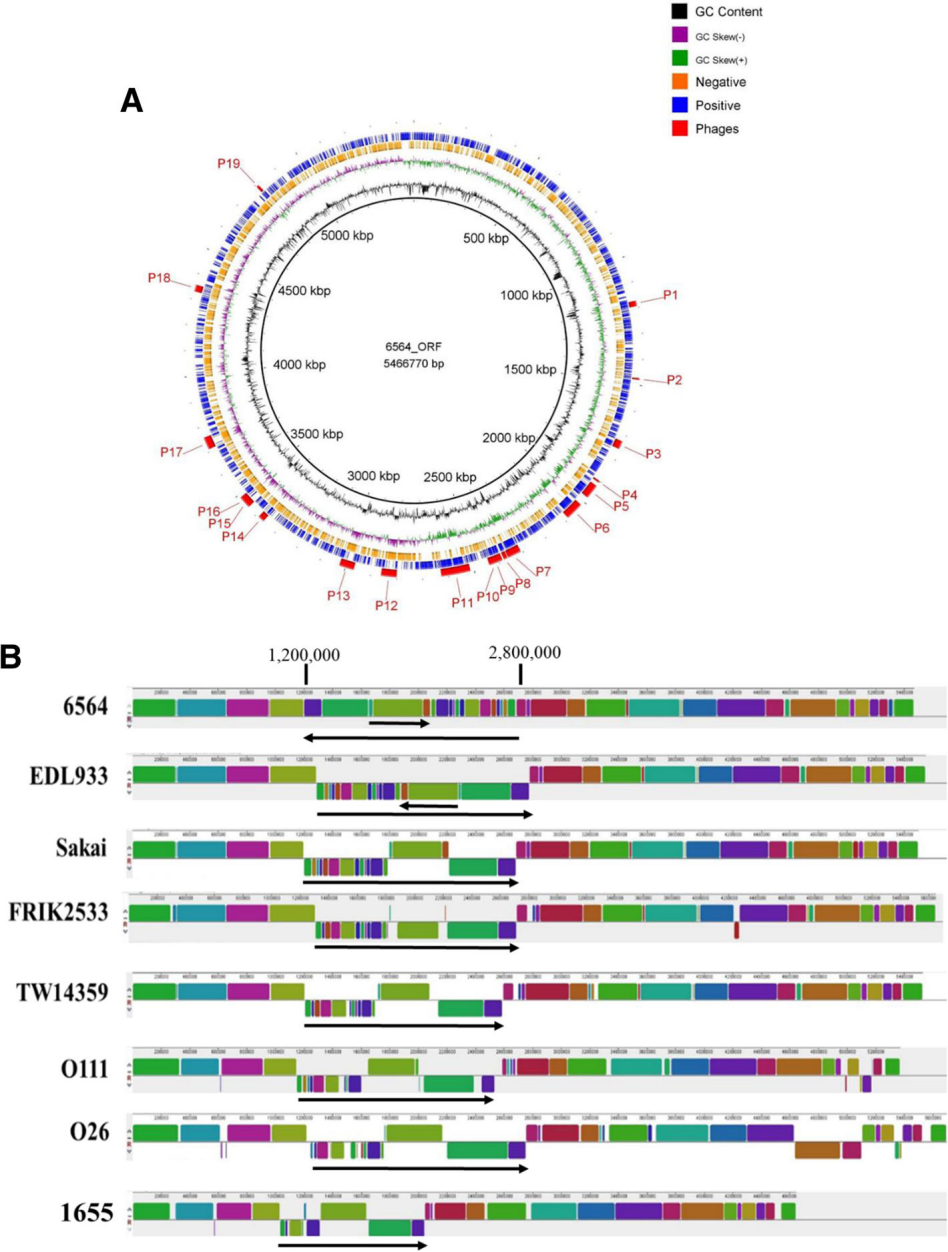
**a** Circular map of the chromosome of *E. coli* O157:H7 strain NADC 6564 generated from its annotated sequence. The map was constructed by downloading the chromosomal sequence into the Blast Ring Image Generator. The legend on the top right corner shows rings representing GC content, GC skew, inserted phage or phage-like elements (red), and ORFs on – (orange) and + (blue) DNA strands. **b** Comparing chromosomal organization of *E. coli* O157:H7 strain NADC 6564 to published chromosomal sequences of other *E. coli* O157:H7 strains (NADC 6565, EDL933, Sakai, FRIK2533, TW14359), non-O157 strains (O111 and O26) and a non-pathogenic *E. coli* K12 strain MG1655. Linear Conservative Blocks or LCB (5–18 when counting from the left) that are underlined with a long black arrow were inverted in their arrangement in strain NADC 6564 compared to the other strains included in this comparison. The LCB 18 and 19 that are underlined with a shorter black arrow had inverse orientation in Sakai compared to the orientation of these two LCB in other strains. Chromosomal sequences were aligned using Mauve

The complete genome of NADC 6564 was sequenced using a combination of PacBio RS II and Illumina MiSeq sequencing methods and assembled and annotated into a chromosome of 5466,770 bp and a plasmid of 92,691 bp as described previously [28]. In the current study, we used BLAST Ring Image Generator (BRIG) to generate a detailed circular map of the annotated NADC 6564 chromosomal sequence (Fig. 1a). This chromosomal map shows predicted protein-coding sequences (CDS) from both its positive and negative strands, which are represented by the two outermost rings (Fig. 1a). These CDS are shown as purple or yellow rectangles, where the size of each rectangle corresponds to the length of the CDS. A total of 19 bacteriophages (labeled P1 – P19) identified in the assembled sequence of NADC 6564 are shown as pink rectangles outside of the positive-chromosomal strand (Fig. 1a).The size of the chromosome, number of CDS, and number of genes encoding rRNA and tRNA were very similar between NADC 6564 and the four O157 reference strains (Table 1). The chromosome of NADC 6564 contained 53 genomic islands (GI) compared to reference strains EDL933, Sakai, and TW14359, containing 63, 71 and 44 GI, respectively (Table 1).

**Table 1:** Comparison of basic features of the strain NADC 6564 chromosome and reference O157:H7 strains

Strains^a^	Size (Mb)	CDS^b^	tRNA genes	rRNA genes	Genomic Islands (GI)	Size range GI (kb)	% GC	Reference	Source
NADC 6564	5.46	5542	103	22	53	4–85	50.0	[28]	Ground Beef
EDL933	5.53	5587	100	22	63	3–88	50.4	[80]	Ground Beef
Sakai	5.49	5504	103	22	71	4–59	50.5	[72]	Sprouts
TW14359	5.52	5555	108	22	44	ND	50.5	[27]	Spinach
EC4115	5.57	5608	109	22	ND^c^	ND	50.5	[25]	Spinach

^a^Accession numbers: CP017251.1 (NADC 6564), CP008957.1 (EDL933), BA000007.2 (Sakai), CP001368.1 (TW14359), and NC_011353.1 (EC4115); ^b^*CDS* Protein-coding sequence, ^c^*ND* Not determined

### Altered organization of conserved genomic blocks in the chromosome of NADC 6564

On the average, the chromosome of O157 strains are about 1.40 Mb larger than that of non-pathogenic *E. coli* K12 strains [47]. All *E. coli* strains share a common core genome of about 4.1 Mb organized into linear conserved blocks (LCB) that are disrupted in pathogenic *E. coli* lineages by the integration of laterally-acquired genetic elements, such as genomic islands (GI) and bacteriophages [47]. These genetic elements carry a wide variety of genes impacting bacterial virulence, metabolism, and genetic organization of chromosomal DNA [25, 27, 72]. In order to determine the basic arrangement and organization of LCB, we compared the Mauve alignment of the whole chromosomal sequence of NADC 6564 to reference O157, and several non-O157 Shiga toxin-producing *E. coli* (STEC) strains. The results of this comparative alignment showed that the chromosomes of compared strains were organized into 40 to 42 LCB (Fig. 1b). These LCB were arranged contiguously from left to right in all compared strains. However, 18 LCB (numbered 5–22 when counting from the left) of strain NADC 6564, which are underlined with a long black arrow, were arranged in an opposite order compared to that in other O157 and non-O157 strains (Fig. 1b). The LCB 18–19 (identified by a short black arrow) had the same orientation in strains NADC 6564 and Sakai compared to that in EDL933 (Fig. 1b).

### Dissimilar types, number, and distribution of bacteriophages and genomic islands in NADC 6564 compared to reference strains

**Fig. 2:**
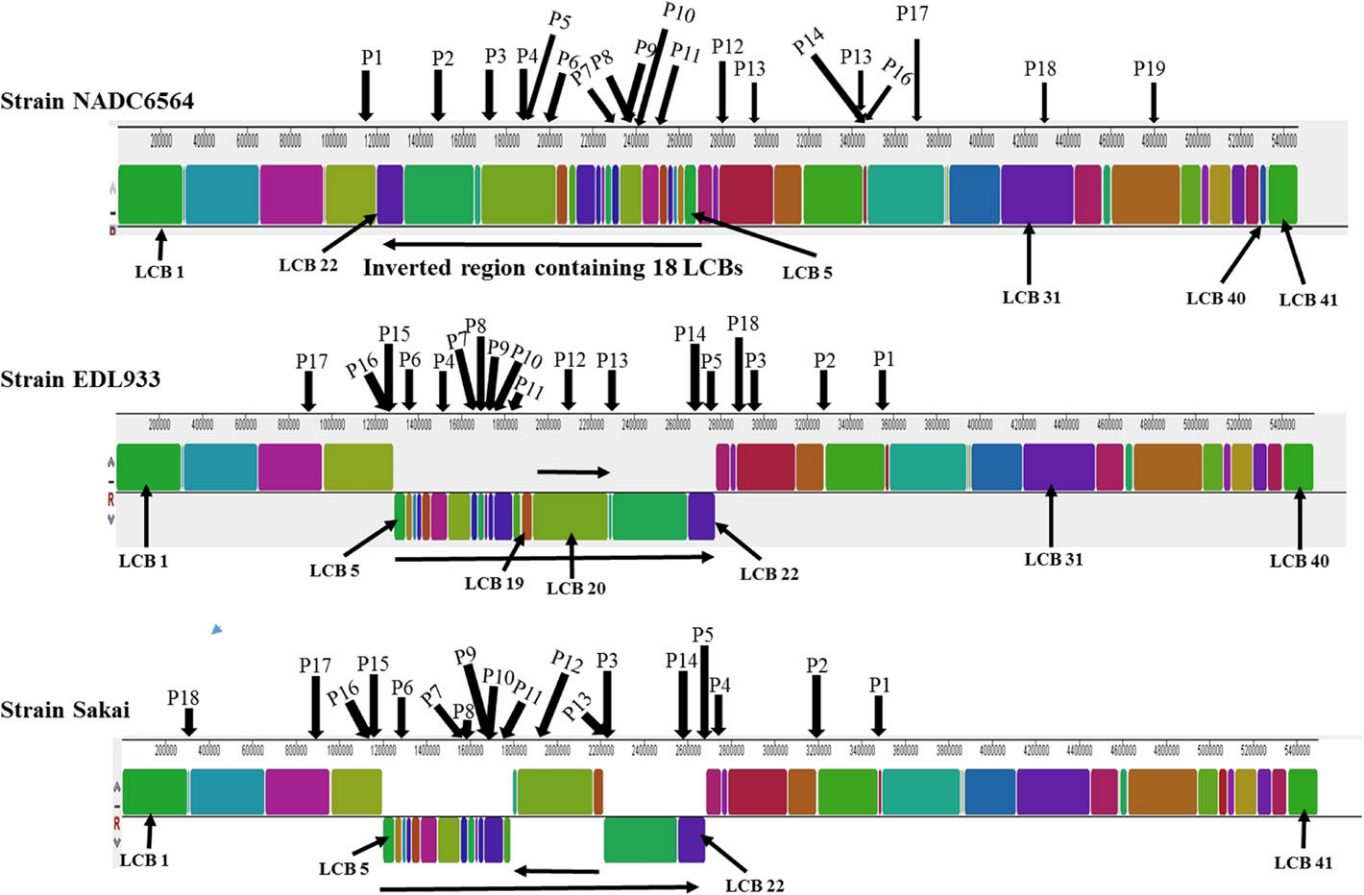
Linear maps showing bacteriophage insertion sites in linear conserved blocks generated by Mauve alignment of the chromosomal sequences of strains NADC 6564 and reference strains EDL933 and Sakai. Phages are numbered from 1 to 19 and arrows indicate sites of phage insertion in LCB

**Fig. 3:**
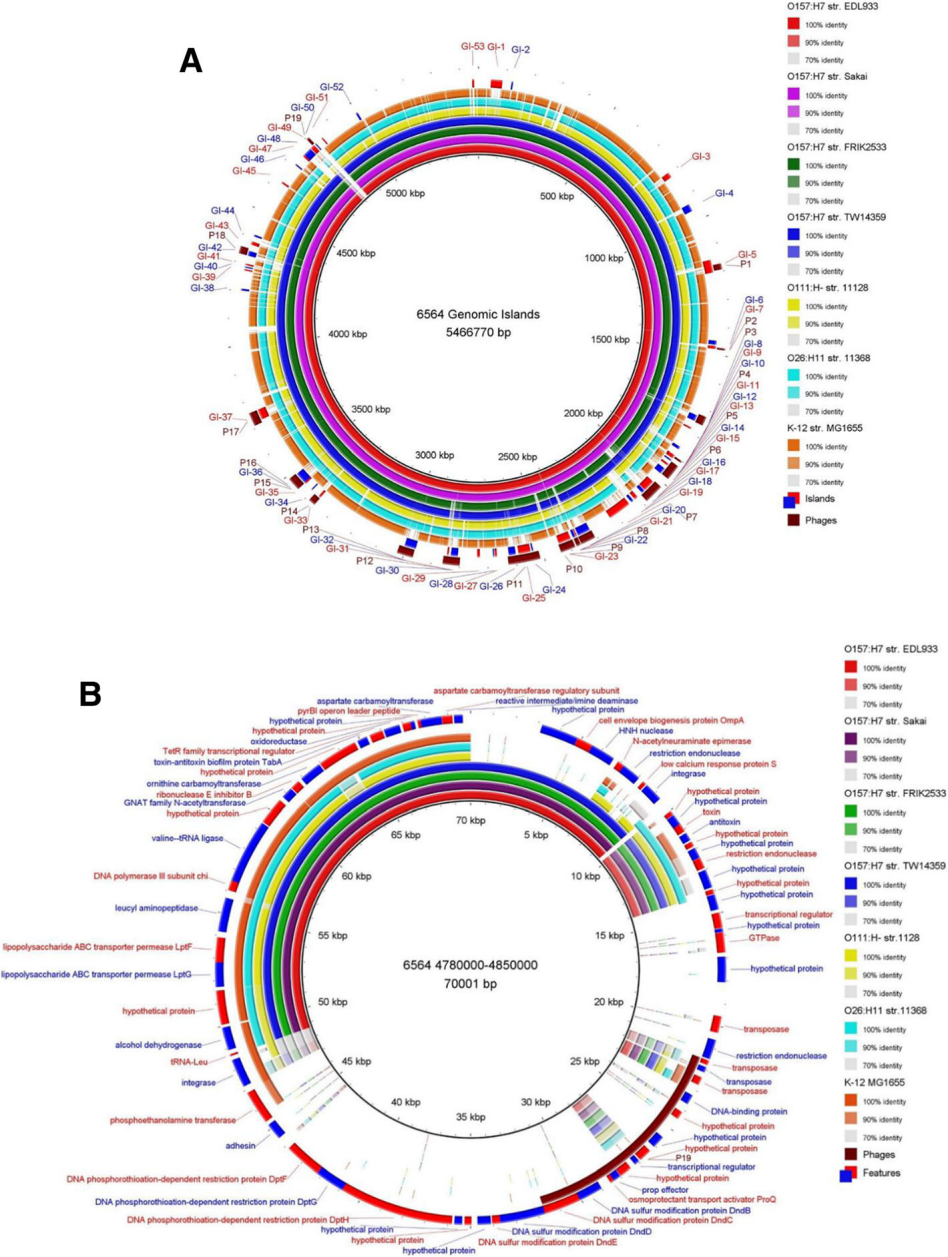
**a** Comparison of Blast Ring Image Generated for *E. coli* O157:H7 strain NADC 6564 (black innermost ring) with similar images generated for other lineage I (EDL933, red ring; Sakai, purple ring), lineage II (FRIK2533, green ring), and lineage I/II (TW14359, blue ring) *E. coli* O157:H7 strains, non-O157 strains (O111, yellow ring; O26, blue ring) and a non-pathogenic *E. coli* K12 strain MG1655 (outermost orange ring). The colored legend to the right shows the degree of nucleotide sequence homology between the sequences of strain NADC 6564 to other O157:H7 and non-O157:H7 *E. coli* strains. The white gaps in rings represent genomic regions that are missing in the corresponding genomes compared to strain NADC 6564. Locations of genomic islands (GI) and phage or phage-like elements (P) are indicated on the two outer rings. **b** Magnified picture of the 39-kb insertion element (IE) identified in the chromosome of *E. coli* O157:H7 strain NADC 6564 but lacking in the chromosomes of other strains listed in the legend on the right side panel. This element is bounded at both ends by a 56 bp repeat sequence (not shown in the figure) and inserted at the tRNA-leu gene in the chromosome. The outer most ring shows relative locations of various genes and their encoded proteins and associated functions

Since arrangement of 18 out of 41 LCB was different in NADC 6564, we wanted to analyze location of laterally acquired genetic elements, such as bacteriophages and genomic islands in NADC 6564 relative to the reference strains. Moreover, the understanding of differences in the chromosomal location of diverse sets of bacteriophages and mobile elements could provide insight into the mechanisms by which these elements differentially impact virulence and other traits of O157 strains [72]. Analysis of the chromosomal sequences by PHASTER [73, 74], a software for identifying phage-like elements in bacterial genomes, resulted in the identification of 19 (P1 – P19) such elements in NADC 6564 (Table 2 and Fig. 2) compared to 18 (P1 – P18) such elements identified in the chromosomes of reference strains EDL933 and Sakai (Additional file 1: Table S2). The locations of these phages are shown outside of the outermost ring of the comparative circular chromosomal map of NADC 6564 and the reference strains created by BRIG [75] (Fig. 3a). The 19 phages of str. NADC 6564 were inserted in different LCB at sites that were either identical or different from the insertion sites occupied by the corresponding similar phages in two reference strains (Fig. 2, Table 2, and Additional file 1: Table S2). For example phage 933 W, which carries the *stx2* gene, occupied the same insertion site that was flanked by the gene (*wbrA*) encoding NAD(P)H: quinone oxidoreductase in str. NADC 6564 and the two reference strains (EDL933 and Sakai). About 10 of the 19 phages in NADC 6564 and the reference strains EDL933 and Sakai are inserted in the region of the genome containing LCB 5–22. The remaining phages were inserted in LCB that are located immediately surrounding LCB 5–22 (Fig. 2 and Table 2). We also analyzed NADC 6564 chromosomal sequence to identify genomic islands (GI) and their locations on this chromosome and compare the results of this analysis with similarly analyzed chromosomal sequences of reference strains. Based on this analysis, we identified 53 GI in the chromosome of NADC 6564 compared to the presence of 63 and 71 GI in the reference strains EDL933 and Sakai, respectively (Additional file 2: Table S3 and Fig. 3a). The overall representation of GI amounted to about 17.08% of the total chromosomal sequence in NADC 6564 compared to about 19.37 and 15.16% for the GI in strains EDL933 and Sakai, respectively. Most of the GI occupied the same LCB in the chromosome of NADC 6564 and the reference strains that had phages integrated in them (Additional file 2: Table S3 and Fig. 3a). This preferential distribution of GI and phages in this chromosomal region (approximately 1000 kb – 3500 kb) indicates a propensity of this region for increased DNA recombination and rearrangements resulting in differential arrangements of LCB in O157 strains.

**Table 2:** Bacteriophage profile of strain NADC 6564

Bacteriophage Region/Name	Sequence Length (kb)	Number of Phage Proteins	Chromosomal Location	Genes Flanking^a^ Phage Insertion Site	GC%	Accession Number
1/Stx2-converting phage 1717	21	7	1,172,843–1,193,884	BHW77_05970 and BHW77_06120	41.96	NC_011357
2/*Shigella* phage Sf6	6.4	4	1,473,190–1,479,623	BHW77_07465 and tRNA-Arg	46.18	NC_005344
3/Enterobacteria phage YYZ-2008	32.1	18	1,723,923–1,756,041	BHW77_08615 and BHW77_08855	52.10	NC_011356
4/Enterobacteria phage BP-4795	6.8	3	1,899,932–1,906,773	BHW77_09510 and BHW77_09560	51.91	NC_004813
5/Enterobacteria phage BP-4795	56.5	14	1,922,013–1,978,603	BHW77_09625 and BHW77_09965	51.37	NC_004813
6/Enterobacteria phage 933 W	70.2	65	2,015,661–2,085,889	BHW77_10140 and BHW77_10630	50.16	NC_000924
7/Enterobacteria phage BP-4795	53.1	24	2,308,130–2,361,308	BHW77_11820 and BHW77_12200	49.29	NC_004813
8/*Brucella* phage BiPBO1	15.2	4	2,364,182–2,379,410	BHW77_12210 and BHW77_12335	51.17	NC_031264
9/*Escherichia* virus Lambda	53.3	29	2,385,028–2,438,396	BHW77_12355 and BHW77_12685	50.26	NC_001416
10/Stx2-converting phage 1717	2.6	3	2,428,442–2,431,075	BHW77_12620 and BHW77_12650	55.88	NC_011357
11/Enterobacteria phage YYZ-2008	113.4	31	2,514,193–2,627,682	BHW77_13055 and BHW77_13845	51.50	NC_011356
12/Enterobacteria phage BP-4795	60	29	2,800,143–2,860,221	BHW77_14595 and BHW77_14980	50.25	NC_004813
13/Enterobacteria phage BP-4795	57.4	32	2,967,268–3,024,670	BHW77_15525 and BHW77_15920	50.94	NC_004813
14/Enterobacteria phage P88	24.8	18	3,361,891–3,386,762	BHW77_17640 and BHW77_17830	50.31	NC_026014
15/*Escherichia* virus Lambda	30.1	14	3,440,475–3,470,640	BHW77_18140 and BHW77_18340	54.39	NC_001416
16/*Shigella* phage POCJ13	24.1	6	3,463,508–3,487,685	BHW77_18270 and BHW77_18470	47.40	NC_025434
17/Enterobacteria phage cdtI	48.2	22	3,709,595–3,757,796	BHW77_19495 and BHW77_19800	50.11	NC_009514
18/Enterobacteria phage SfI	26.1	6	4,330,960–4,357,158	BHW77_22420 and BHW77_22605	45.57	NC_027339
19/Acidianus tailed spindle virus	10.1	1	4,801,573–4,811,702	BHW77_24685 and BHW77_24755	46.57	NC_029316

^a^Genes flanking the phage insertion sites are represented as locus-tags in the annotated sequence (Accession number: CP017251.1)

### Relationship of strain NADC 6564 to lineage I versus the other lineages

O157 is considered an emerging foodborne pathogen presumably due to reported genetic heterogeneity of O157 populations enabling them to diverge into isolates that differ in their abilities to colonize the carrier host animal, cause disease in humans, or survive in the external environment [33–35, 76]. Population heterogeneity is driven by intrinsic genetic changes, such as point mutations, small DNA insertions or deletions, as well as through the lateral acquisition of genomic islands and bacteriophages [25, 30, 72]. Since genome heterogeneity has been used for classifying O157 strains into three major evolutionary lineages I, I/II, and II [21, 45], we performed comparative BLAST analysis to classify the lineage of NADC 6564. Figure 3a summarizes the comparative BLAST homologies of NADC 6564 to O157 strains of lineage I, II, and I/II, to non-O157 Shiga toxin-producing *E. coli* strains, and to a non-pathogenic *E. coli* K12 strain MG1655. As shown in Fig. 3a, greatest homology was observed between the chromosomal sequence of NADC 6564 and the lineage I O157 strains (EDL933 and Sakai) because only a few regions (4–5), each consisting of a short stretches of nucleotides, were missing in strains EDL933 and Sakai compared to NADC 6564. The chromosomes of lineages II (FRIK2533) and I/II (TW14359) O157 strains were lacking 24 to 26 regions of variable lengths that were present in NADC 6564 (Fig. 3a). The non-O157 Shiga toxin-producing *E. coli* O26 and O111 strains had the highest number of regions missing in their chromosomes compared to NADC 6564 and other O157 strains irrespective of their lineage affiliation (Fig. 3a).

### BLAST results identified a novel 39-kb insertion element in NADC 6564

Despite extensive whole chromosome sequence homology between NADC 6564 and other O157 strains of lineage I, as revealed by BLAST analysis, a 39-kb (4,789,473 bp – 4828,646 bp) region carrying bacteriophage P19 was present only in the chromosome of NADC 6564 and was missing from chromosomes of all other O157 and non-O157 strains (Fig. 3b). This 39-kb insertion element (IE) is bounded by a 56-bp direct repeat at its 5′ (4,789,473 bp – 4,789,529 bp) and 3′ (4828,590 bp – 4828, 646 bp) ends and is inserted near the gene encoding for tRNA-Leu. BLAST analysis confirmed that none of the O157 and non-O157 strains, whose chromosomal sequences were available in the GenBank, contained this 39-kb IE. However, this IE was present in the unpublished genomes of *E. coli* strain AR0015 (Accession Number: CP024862.1) and *E. coli* strain C1 (Accession Number: CP010116.1). The genome of a porcine *E. coli* strain PCN061 contained two regions, one 23 kb and the other 7.1 kb in size, that were homologous to the 39-kb IE with only 1.0 kb non-homologous DNA separating the two homologous regions [77].

### The 39-kb IE encodes genes for mobility, potential virulence, and stress tolerance in strain NADC 6564

As shown in Fig. 3b, the 39-kb IE of strain NADC 6564 encodes for an integrase located near its 5′ end, which is inserted near the tRNA-Leu gene. The 3′ region of this IE also encodes a transposase (in addition to those encoded on P19), GTPase, and a transcriptional regulator in the 3′ region. The presence of genes encoding an integrase and a transposase, and the presence of a 56-bp direct repeat at the ends, are all suggestive that the 39-kb DNA region is an insertion element acquired independently by NADC 6564. In addition, the 39-kb IE also contains genes encoding for a phosphoethanolamine transferase, adhesion, and *dptFGH* and *dndBCDE* operons predicted to encode DNA phosphorothioation or (S-DNA modification) and a restriction system (Table 3) for recognizing and degrading DNA lacking the S-DNA modification, respectively [57, 58]. However, the *dndA* gene, which represents the fifth gene of the *dnd* operon (*dndABCDE*) in many bacterial species [59], is lacking in the genome of NADC 6564. In *E. coli* and many other bacterial species that contain only four- (*dndBCDE*) instead of five-gene *dnd* operon (*dndABCDE*), the *iscS* gene represents an ortholog of the *dndA* gene [78, 79]. IscS functions as a cysteine desulfurase which is a key enzyme of DNA phosphorothioation pathway [79]. The NADC 6564 does encode *iscS* which occupies 1,289,336-1,290,550 bp in its genome. Although genes showing 100% homology to the adhesin gene encoded in the 39-kb IE of NADC 6564 are present in extraintestinal *E. coli* strain PCN061 [77], avian pathogenic *E. coli* strain O1 (GenBank Accession Number: CP028310.1), uropathogenic *E. coli* strain UT189 (GenBank Accession Number: CP000243.1), and enterohemorrhagic *E. coli* strain 104:H21 [80], the contribution of this adhesin to virulence of these strains has not been fully described.

**Table 3:** Specific genes and their functions encoded in the 39-kb insertion element

Genes	Proteins/enzymes	Functions
*peaX* ^a^	Phosphoethanolamine transferase (PEAX)^a^	LPS modification/colistin resistance
*dndBCDE*	DNA sulfur modification proteins^b^	DNA phosphorothioation (replacing non-bridging phosphate oxygen with sulfur in the DNA backbone)/oxidative stress response
*dptFGH*	DNA phosphorothioation-dependent restriction enzymes^a^	DNA restriction of phosphorothioated DNA/foreign DNA recognition and restriction
*proQ* Other genes	ProQ Adhesins, DNA-binding proteins, transposases, hypothetical proteins	Osmoprotectant transport activator Possible gene expression regulation, DNA recombination, and unknown functions

^a^PEAX represents phosphoethanolamine transferase encoded by the *peaX* gene located on the 39-kb IE of NADC 6564

^b^Activities of these enzymes were verified by specific in vitro assays as described in Methods and Results

### The *dndBCDE* operon conferred S-DNA modification that protected modified DNA from restriction activity encoded by the *dptFGH* operon

**Fig. 4:**
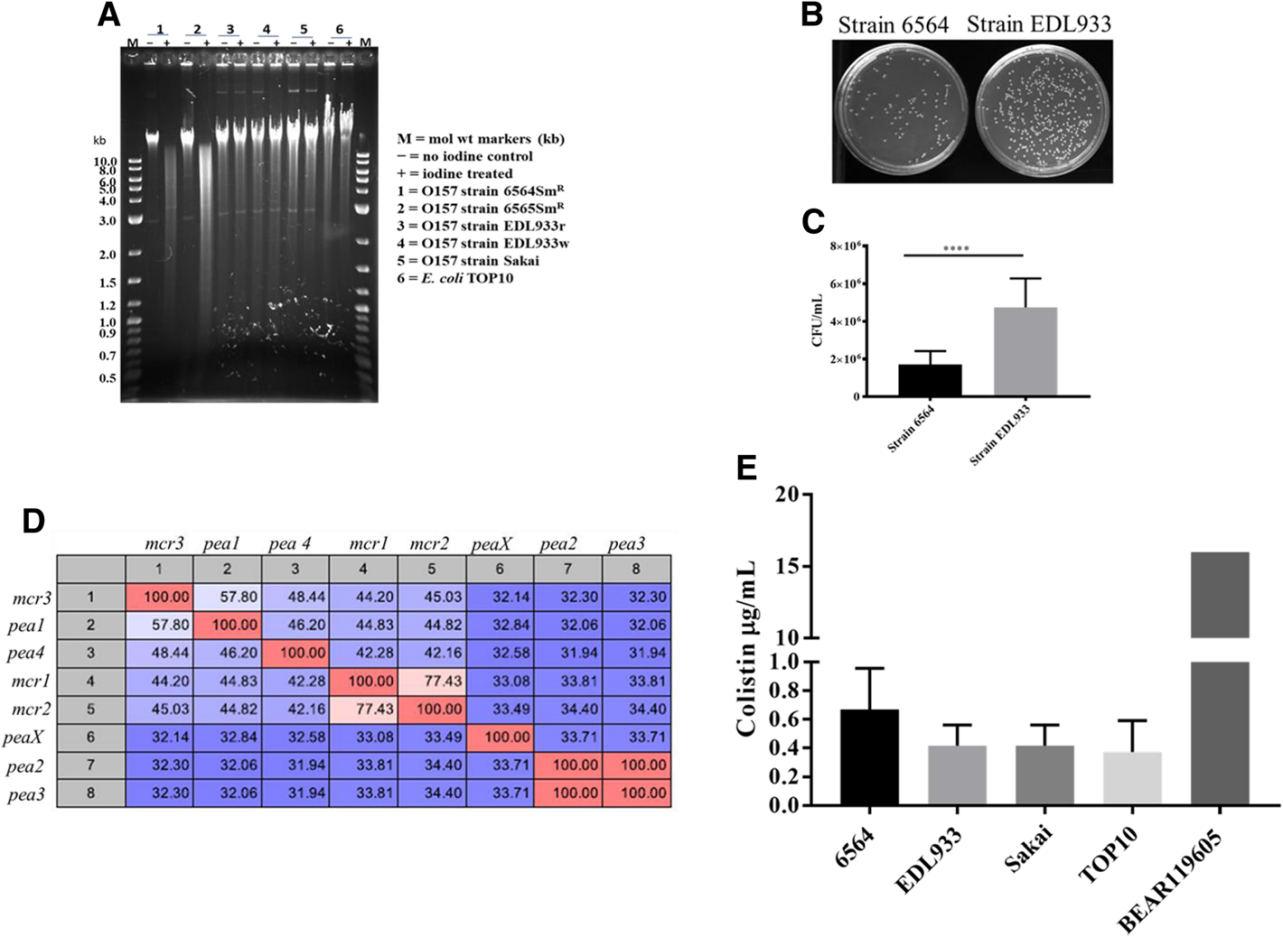
Confirming DNA phosphorothioation and DNA restriction activities encoded by *dndBCDE* and *dptFGH* operons. **a** Equal amounts of genomic DNA isolated from NADC 6564 and other strains listed on the right side of the gel picture was either treated or not treated with iodine. The treated DNA was loaded in to lanes marked with + and untreated DNA loaded in to lanes marked with – signs. Smearing effect in the lane loaded with iodine-treated DNA is indicative of DNA degradation compared to the presence of intact DNA (approximately 25 to 50 kb in size based on the size of molecular weight markers loaded in lanes marked M) in lanes loaded with untreated DNA. **b** and **c** Confirmation of S-DNA modification-dependent restriction activity encoded by the *dpt* operon. The pUC18 plasmid DNA lacking S-DNA modification was electroporated into strains NADC 6564 (*dpt* operon-positive) and EDL933 (*dpt* operon-negative) and recovery of this plasmid was determined by comparing number of colonies produced on LB agar-carbenicillin (100 μg/mL) by the electroporated cultures of these strains (**b**). Absolute colony counts shown represent mean plus and minus standard deviation of three independent experiments (**c**). **d** Table showing comparisons of nucleotide sequence homology of *pea*X gene encoded on the IE to other four *pea* genes located elsewhere on the chromosome of NADC 6564 and to *mcr* genes encoding colistin resistance in *E. coli*. Numbers in colored checker boxes represent homology between compared genes numbered 1–7 and labeled with corresponding gene name on the outer margins of the Table. **e** Graph showing the plot of minimum inhibitory concentration (MIC) of colistin in strain NADC 6564 compared to strains EDL933 and Sakai lacking the *pea*X gene and the 39 kb insertion element. *E. coli* TOP10 (sensitive to colistin) and *E. coli* BEAR119605 (resistant to colistin) were used as negative and positive controls in the MIC assay. Results are mean plus the standard deviation of three independent assays. **** = *p* < 0.0001

The function of the *dndBCDE* operon encoded by the 39-kb IE in NADC 6564 and NADC 6565 (a Congo red-positive variant of NADC 6564 included as a control) [28] was verified by demonstrating that the genomic DNA of these two strains was susceptible to degradation by an iodine solution compared to no effect of this treatment on the genomic DNA of O157 strains EDL933 and Sakai, and non-pathogenic *E. coli* TOP10 lacking this operon (Fig. 4a). These results indicated that genes in the *dndBCDE* operon are expressed and their encoded proteins are able to S-modify DNA in NADC 6564. Similarly, we also demonstrated that the presence of *dptFGH* operon resulted in significantly (*p* < 0.05) lower recovery of colonies on agar plates that were surface-spread with NADC 6564 transformed with plasmid pUC19 DNA lacking S-modification compared to higher number of colonies recovered from O157 EDL933 transformed with same plasmid DNA (Fig. 4b and c). These results indicated that the *dptFGH* operon in NADC 6564 encodes for a restriction system capable of restricting incoming heterologous DNA (pUC19) devoid of S-DNA modification compared to strain EDL933 lacking the *dptFGH* operon. However, we did not investigate whether transforming strains NADC 6564 and EDL933 with S-DNA-modified pUC19 (recovered from pUC19-transformed NADC 6564) or transforming NADC 6564 *dptFGH* deletion mutant and EDL933 with pUC19 would show equivalent recovery of the transformed DNA, respectively, to rule out differences in the transformation efficiency of these two strains. Although the *dndBCDE* and *dptFGH* operons are widely distributed among diverse groups of bacterial species, including pathogenic and non-pathogenic *E. coli* [56–58], BLAST analysis of *dndBCDE* (4,808,986 bp– 4,814,048 bp; Accession Number CP017251.1) and *dptFGH* (4,815,824 bp – 4,823,838 bp, Accession Number CP017251.1) sequences against the nucleotide sequences in GenBank did not retrieve any O157 strains carrying these genes. However, sequences showing 99% homology to the DNA regions carrying *dndBCDE* and *dptFGH* operons of NADC 6564 were identified in a porcine *E. coli* strain PCN061 [77] and two other unpublished genomes of *E. coli* strains (AR0015: Accession Number CP024862.1; AR436: Accession Number CP029111.1). The sequences exhibiting homology ranging from 78 to 81% to nucleotide sequences of the *dndBCDE* and *dptFGH* operons of NADC 6564 were also identified in *Salmonella enterica* (Accession Number: CP019412.1), *Enterobacter* sp. 638 (Accession Number: CP000653.1), *Yersinia ruckeri* str. YRB (Accession Number: CP009539.1), and *Erwinia amylovora* str. E2 (Accession Number: CP024970.1). An evolutionary tree generated by comparing the homology of genes in the *dndBCDE* (Additional file 3: Figure S1) and *dptFGH* (Additional file 4: Figure S2) operons to bacterial nucleotide sequences in GenBank revealed that NADC 6564 might have acquired these genes from a progenitor *E. coli*-type strain that produced two earlier clades of *E. coli* carrying *dndBCDE* and *dptFGH* operons. Later acquisition of *dndBCDE* and *dptFGH* operons by other bacterial species in Enterobacteriaceae produced multiple clades over the course of the evolution of these bacterial species.

### Phosphoethanolamine transferase encoded on the 39-kb IE showed poor homology to other phosphoethanolamine transferases

The phosphoethanolamine transferases (PEA) are a family of proteins that mediate lipid A modification, and some PEA proteins encoded by *mcr* genes confer high levels of colistin resistance in *E. coli* [61]. Besides the presence of a phosphoethanolamine transferase (referred to as PEAX encoded by the sequence 4,825,365 bp – 4,826,864 bp) on the 39-kb IE, homology analysis revealed that NADC 6564 contains four additional phosphoethanolamine transferases (PEA) encoded at different regions (PEA1: 2,157,481 bp – 2,159,100 bp; PEA2: 217,878 bp – 219,569 bp; PEA3: 3,672,079 bp – 3,673,662 bp; and PEA4: 4,985,684 bp – 4987, 327 bp) of its chromosome. The reference strains EDL933 and Sakai carry three and four genes encoding for PEA in their chromosomes, respectively. The amino acid sequence homology comparison revealed that among the five phosphoethanolamine transferases (PEA1, PEA2, PEA3, PEA4 and PEAX) of strain NADC 6564, PEA1 and PEA4 shared higher homology between their amino acid sequences (46% identity). The amino acid sequence of PEA1 also showed higher homology to the amino acid sequences of PEA transferase encoded by *mcr3* (58%) and *mcr1* and *mcr2* (45%) (Fig. 4d). On the other hand, amino acid sequences of PEA3, PEA4, and PEAX showed lower homology to each other (32–34% identity) and to the amino acid sequences of PEA encoded by the three *mcr* genes (Fig. 4d). Since one of the phosphoethanolamine transferase (PEAX) is encoded by the 39-kb IE, we compared the nucleotide sequence of the *peaX* ORF (1499 bp) against the GenBank database to determine its homology to the nucleotide sequences of *pea* ORFs of *E. coli* strains and other bacterial species. The results of this analysis showed that *peaX* ORF was highly homologous to *pea* ORFs from extraintestinal pathogenic *E. coli* strains PCN033 and PCN061 (100%) [77], avian pathogenic *E. coli* strain O1 (99%) (GenBank Accession Number: CP028310.1), uropathogenic *E. coli* strain UT189 (99%) (GenBank Accession Number: CP000243.1), and enterohemorrhagic *E. coli* strain 104:H21 (99%) [80]. The minimum inhibitory concentration (MIC) of colistin for NADC 6564 containing five phosphoethanolamine transferases was slightly but insignificantly (*p* > 0.05) higher (0.66 μg/mL) compared to the colistin MIC (0.41 μg/mL) in reference strains (EDL933, Sakai, and a non-pathogenic *E. coli* TOP10) carrying three to four phosphoethanolamine transferases (Fig. 4e). Whether the small increase in colistin resistance of NADC 6564 was due to the presence of the additional phosphoethanolamine transferase (PEAX) encoded by the 39-kb IE was not determined because overall NADC 6564 still remained colistin sensitive like the reference strains. A phylogenetic tree constructed by comparing the homology of genes (*pea1*, *pea2*, *pea3*, *pea4*, and *peaX*) encoding five phosphoethanolamine transferases of NADC 6564 to genes encoding phosphoethanolamine transferases in other bacterial species revealed that all five *pea* genes shared a common ancestor but diverged independently into separate clades over time (Additional file 5: Figure S3).

### Comparative genomics facilitated inferring the evolutionary relationship of NADC 6564

**Fig. 5:**
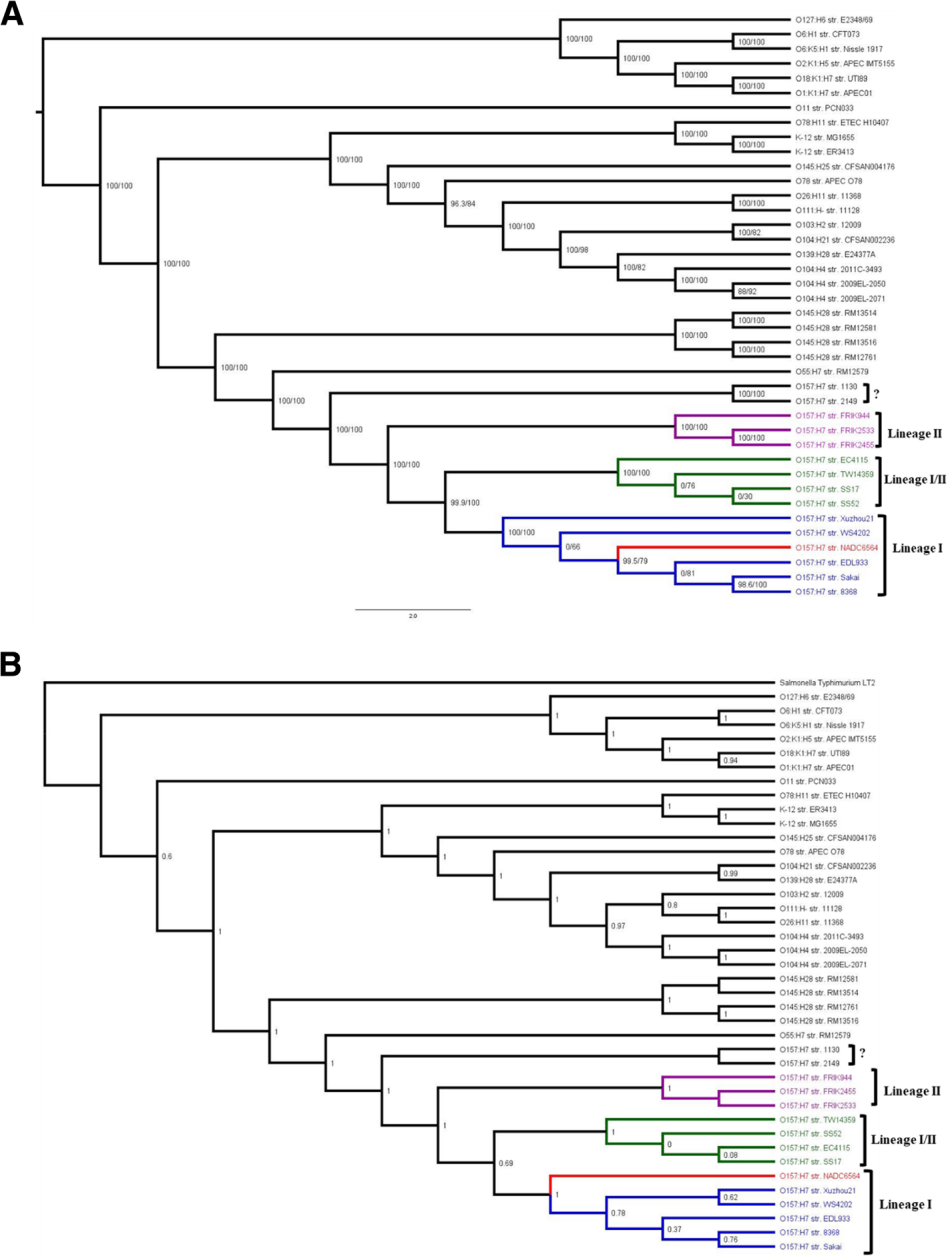
Cladograms showing evolutionary relationship of *E. coli* O157 strain NADC 6564 to other pathogenic O157 and non-O157 strains. **a** Cladogram produced by constructing concatenated core genomes from whole genome sequences of 40 bacterial strains listed in Additional file 6: Table S1. A total of 2284 core genes were identified among the 40 strains used for constructing core genomes. These core genomes were used to infer a maximum likelihood phylogenetic tree using IQTREE. The generated tree was visualized using FigTree. Since there was no outgroup included in the tree, FigTree’s midpoint rooting method was used to root the tree. **b** Cladogram inferred using the parSNP-Gingr pipeline. Whole genomes for the sequences being compared were provided as input to parSNP using the NADC 6564 annotated GenBank file as the reference. Evolutionary trees were created by visualizing the parSNP output in Gingr

In order to infer the evolutionary relationship of NADC 6564 to other O157 and non-O157 strains, we constructed two types of maximum-likelihood trees using IQ-TREE and different sets of data as input. To generate the first tree, we selected 2000 genes from 40 different pathogenic and non-pathogenic *E. coli* strains to construct a concatenated core genome representative of these strains. The evolutionary tree generated by using the concatenated core genome (Fig. 5a) grouped O157 strains into four clades that are colored blue (clade1), green (clade 2), purple (clade 3), and black (clade 4). The strain NADC 6564 (shown in red font) grouped with clade1 O157 strains. This grouping agreed with the previously reported evolutionary groupings of O157 strains using octamer-based genome scanning [21]. To further confirm the validity of the tree shown in Fig. 5a, we also constructed an evolutionary tree by comparing single nucleotide polymorphism (SNP) profiles generated for the whole genome sequences of O157 and non-O157 strains using annotated genome of NADC 6564 as a reference. The tree generated from SNP profiles comparison assigned O157 strains into four groups (Fig. 5b), and this grouping was similar to that generated for O157 strains using a concatenated core genome as a reference (Fig. 5a). However, the evolutionary tree shown in Fig. 5b suggested that NADC 6564 might have diverged much earlier from other lineage I O157 strains included in this tree.

## Discussion

*Escherichia coli* O157:H7 (O157) has become a prominent foodborne pathogen not only in North America but world-wide since the first reported association O157 with human disease in early 1980s [12, 81]. O157 has been called an emerging foodborne pathogen due to the continuous evolution of its genome via acquisition of laterally transferred mobile DNA elements and other mutagenic events [26, 30, 45, 72]. Many of the newly emerged clones of O157 that have caused outbreaks inflicting high morbidity and mortality in infected individuals have in part been linked to increased virulence of the implicated O157 strains [27]. Thus, understanding genomic diversity and plasticity of O157 strains is important for determining epidemiology, understanding bacterial pathogenesis, identifying specific biomarkers, predicting severity of the disease, tracing origin of these strains, and developing vaccines for controlling these pathogens in host reservoirs.

In a previous study, we reported basic features of the genome of a foodborne isolate of O157 strain 86–24 linked to the 1986 human disease outbreak [28]. In the present study, we used comparative genomics to analyze chromosomal sequence of this isolate, named NADC 6564, to determine its genetic content and organization, unique functional attributes, genetic lineage, and evolutionary relationship to other well-characterized O157 strains. The genetic contents, in terms of the total nucleotides constituting the chromosome of NADC 6564 (5.46 Mb), was slightly smaller than the reference strain O157 EDL933 (5.53 Mb) and O157 Sakai (5.49 Mb). Although all *E. coli* share about 4.1 Mb core genome sequence, in pathogenic *E. coli* strains, such as Shiga toxin-producing *E. coli* (STEC) O157:H7 and non-O157 STEC strains, the core genome is disrupted by the insertion of laterally-acquired mobile DNA elements. The acquisition of variable numbers and types of insertion elements, such as bacteriophages (phages), transposons, and genomic islands (GI), is one of the major factors in genome size variability among different O157 and non-O157 strains [47, 72, 82]. For example, screening of the whole chromosomal sequence for inserted phages and GI revealed the presence of 19 phages (named P1 – P19) and 53 GI in NADC 6564 compared to the presence of 18 phages and 63 GI in reference strain EDL933 and 18 phages and 71 GI in the reference strain Sakai. These laterally-acquired elements would account for about 1.645 Mb of DNA in NADC 6564 compared to 1.641 Mb and 1.372 Mb of acquired DNA in the two reference strains EDL933 and Sakai, meaning that these strains would have 3.815 Mb, 3.889 Mb, and 4.118 Mb, respectively of core *E. coli* DNA as part of their genomes. The reported core *E. coli* DNA for the two reference O157 strains is about 4.1 Mb [83].

A variety of high resolution molecular subtyping methods have been developed for discriminating O157 strains and inferring their evolutionary relationships. Some of these methods have classified O157 strains into three lineages, human-biased lineage I strains, bovine-biased lineage II strains, and lineage I/II strains, which are ambiguous with respect to host association and phenotype [21, 45, 84]. We used available bioinformatics tools to construct evolutionary trees based on concatenated core genome and SNPs of NADC 6564 and the reference O157 strains. In these two trees, str. NADC 6564 and the reference O157 strains were grouped correctly, which was in agreement with previous studies [21, 45, 84], into lineage I, II, and I/II strains. However, in the tree constructed by comparing concatenated core genome, NADC 6564 appeared to be closely related to lineage I strain EDL933 as these two shared a common ancestral strain. In the tree generated by comparing SNP profiles that took into consideration 58,505 SNPs, NADC 6564 is more distantly related to the lineage I reference strains (EDL933, Sakai, XuZhou21, WS4202, and str. 8368). Since phylogeny deduced by comparing SNP profiles can discriminate closely related bacterial genotypes based on their microevolutionary history [30], it is possible considering differences in SNP profile of NADC 6564 from other lineage I O157 strains that either NADC 6564 diverged from other lineage I strains or vice versa over time. The identification of a unique 39-kb IE, altered chromosomal organization, and a distinctive phage profile provide important supporting evidence that the NADC 6564 might also have a distinctive SNP profile compared to the other lineage I strains, and warrant characterization of these SNP differences in future studies.

Besides observing subtle differences in the total amount of acquired DNA between NADC 6564 and reference strains EDL933 and Sakai, the organization of the core chromosomal sequence of strain NADC 6564 differed strikingly from the reference strains. We found that 1.4 Mb of the genome represented by contiguous 18 linear conserved blocks or LCB were inverted in their orientation in the chromosome of strain NADC 6564 compared to reference O157 and non-O157 strains. This disparate organization of the large portion of the chromosome would suggest occurrence of specific recombination events presumably resulting in the inversion of this large section of the chromosomal sequence in NADC 6564. Since the laterally-acquired DNA elements in O157 strains are or were mobile elements, the differential integration and subsequent recombination events, such as truncations and excisions, in the chromosome could account for observed variations in the arrangement of conserved core genome blocks among O157 strains [72, 82, 85].

The laterally-acquired elements most often carry genes that could impact the virulence attributes of bacterial strains, and this phenomenon is well exemplified in emergence and evolution of O157 and non-O157 STEC strains. It has been proposed that STEC O157:H7 strains evolved from an ancestral strain similar to enteropathogenic *E. coli* (EPEC) O55:H7 that had already acquired a locus of enterocyte effacement (LEE) for adherence to and colonization of human intestinal tract [52, 53]. This LEE-carrying ancestral strain in the distant past diverged into EPEC O55:H7 and STEC O157:H7 lineages. STEC O157:H7 lineage resulted from lateral acquisition of Shiga toxin-encoding bacteriophages, new genomic islands (GI) encoding O157-specific O antigens, and several other GI and bacteriophages impacting virulence and metabolic repertoire of STEC O157:H7 isolates [52, 53, 72, 82]. Whole genome sequence analysis of NADC 6564 confirmed the presence of both the Shiga toxin 2-encoding bacteriophage (numbered P6; Fig. 2) and LEE-encoded pathogenicity island inserted in the vicinity of GI 15 and GI 3, respectively. The Stx2 phage and LEE were inserted in the chromosome of NADC 6564 adjacent to *wbrA* and tRNA-Sec, respectively. These two elements are also inserted at the same sites in EDL933 and Sakai strains [86, 87]. In addition, we identified a 39-kb DNA region bearing features of an insertion element (IE), such as the presence of a 56-bp direct repeat at its ends, genes for integrase and transposases, a phage remnant (labeled P19), and use of tRNA-leu integration site in the NADC 6564 chromosome. This 39-kb IE is inserted in LCB 31, and based on BLAST homology search none of the O157, non-O157, and nonpathogenic *E. coli* K12 used as reference strains harbored this IE in their genomes (Fig. 2). However, genomic regions ranging in size from 30 to 37 kb, showing 100% homology to the corresponding regions of the 39-kb IE of NADC 6564, were identified in a porcine *E. coli* strain PCN061 [77] and unpublished genomes of *E. coli* strain AR0015 (Accession Number: CP024862.1) and *E. coli* strain C1 (Accession Number: CP010116.1). Most other *E. coli* strains showed only a limited homology (50% or less) to the 39-kb IE of NADC 6564. These results suggest that a few *E. coli* strains including NADC 6564 had acquired this IE through lateral gene transfer while the other *E. coli* strains either had not acquired or had this IE deleted during the course of evolution.

Besides carrying genes for integrase and transposases, the 39-kb IE of NADC 6564 carried a 10.1 kb phage remnant and genes encoding for an adhesin, phosphoethanolamine transferase, S-DNA modification and a DNA restriction system that could impact virulence and survival of NADC 6564. For example, the homologs of the adhesin gene present on the 39-kb IE of NADC 6564 were identified in *E. coli* PCN061 of porcine origin [77], uropathogenic *E. coli* strain UT189 (Accession number CP000243.1), and enteroaggregative *E. coli* strain O104:H21 that was linked to a major disease outbreak in Europe in 2011 affecting 4321 people, of which 852 developed hemorrhagic uremic syndrome and 53 deaths [88].

The phosphoethanolamine transferases are a family of proteins that mediate outer membrane lipid A modification and some members of this family that are encoded by the *mcr* genes confer high levels of colistin resistance in *E. coli* [61]. It has been suggested that the modification of lipid A by the chromosomally encoded phosphoethanolamine transferases could result in a slight increase in the minimum inhibitory concentration (MIC) of colistin and other cationic antibiotics that could confer survival advantage to *E. coli* O157:H7 in certain environmental niches [62]. We were able to demonstrate that MIC of colistin in NADC 6564 containing genes (*pea1*, *pea2*, *pea3*, *pea4*, and *peaX*) encoding five phosphoethanolamine transferases (PEA1 – PEA4 and PEAX), was slightly elevated compared to the reference strains containing genes encoding only three or four phosphoethanolamine transferases. Based on evolutionary tree analysis of *pea* and *peaX* genes (Additional file 5: Figure S3), the *peaX* gene (encoded by the 39-kb IE) of NADC 6564 might have evolved in an *E. coli-*type ancestral strain and subsequently acquired by O157 strains, such as NADC 6564, very early in their evolution compared to the acquisition of other *pea* genes by these strains. Evolutionary tree data and the observed low homology of *peaX* gene to other *pea* genes suggest that other *pea* genes have undergone greater divergence in their nucleotide sequences during the course of evolution compared to the *peaX* gene since their divergence from the first common ancestral strain.

The *dndBCDE* and *dptFGH* operons located on the 39-kb IE in NADC 6564 were functionally active as both S-DNA modification and restriction of DNA lacking S-DNA modification were observed. The *dndBCDE* and *dptFGH* operons are widely distributed in diverse bacterial species [57], but we for the first time have identified these operons in an O157 strain. In addition, demonstration of the presence of *dnd* genes in genomic islands in a majority of *dnd-*carrying *E. coli* strains, including NADC 6564 as we have described in the current study, indicates the importance of these islands in horizontal transfer of *dnd* genes [58]. Many bacterial species possess both *dndBCDE* and *dptFGH* operons organized in a same order but some bacterial species only harbor the *dndBCDE* genes [57, 89]. Since the *dndBCDE* operon has been shown to confer protection to bacterial hosts from a variety of stressors, especially oxidative and high temperature stressors through S-modification of DNA [56], it is feasible that NADC 6564 carrying these genes might have better survival advantages under conditions of high oxidative stress that could be induced in the gastrointestinal tract of the host reservoir animal or in the gastrointestinal tract of infected humans by a variety of factors. Evolutionary tree analysis of *dndBCDE* and *dptFGH* operons present in diverse bacterial taxa appeared to suggests that NADC 6564, like many other *E. coli* strains that it clusters with [58], might have acquired these genes very early in the evolution and further dissemination of *dndBCDE* and *dptFGH* genes occurred later to other bacterial species (Additional file 3: Figure S1; Additional file 4: Figure S2).

## Conclusion

In summary, we have utilized comparative genomics to provide insights into the genetic and functional organization of the whole chromosomal sequence of a foodborne isolate of *E. coli* O157:H7 strain NADC 6564. By doing so, we discovered not only a unique phage distribution profile and a novel insertion element encoding a set of functions relevant to enhanced stress tolerance, but also identified some disparities in its evolutionary relationships with other closely related lineage I strains. Characterization of the SNP profile and SNP effects on specific phenotypes of NADC 6564 would provide a greater understanding of the microevolutionary history behind the observed evolutionary relationship of NADC 6564 to other lineage I strains. In addition, studying the effects of SNPs on gene expression and determining whether the acquired stress related functions confer host and/or environmental fitness would help predict virulence potential and ability to survive in growth-limiting environments.

## Methods

### Construction of circular chromosomal maps

The chromosomal maps of bacterial strains were created and compared to each other utilizing the BLAST Ring Image Generator (BRIG). BRIG uses BLAST to map homologous regions from the query sequences to a circular map of the reference sequences [75, 90, 91]. The annotated chromosome of *E. coli* O157:H7 NADC 6564 [28] was used as a reference for generating whole chromosomal sequence comparisons with selected query sequences. BRIG was also used to generate detailed maps of the regions that were uniquely identified in NADC 6564 but were absent in query sequences. The names and accession numbers of sequences used in this study were downloaded from the NCBI database (Additional file 6: Table S1).

### Identification of genomic islands, phage or phage-like elements, and DNA repeats

The location of putative phage or phage-like regions in the chromosome of NADC 6564 were determined by downloading a FASTA file of the whole chromosomal sequence of this strain from GenBank and then uploading this file to PHASTER [73, 74]. To determine if phage regions identified in NADC 6564 have homologous counterparts in reference strains EDL933 and Sakai, we BLAST searched NADC 6564 phage regions against the chromosomal sequences of these two reference strains. Genomic islands (GI) in NADC 6564 were predicted using the online IslandViewer4 [92]. IslandViewer4 integrates the IslandPath-DIMOB, SIGI-HMM, and IslandPick prediction methods to find probable locations of GI. Since multiple methods are integrated by IslandViewer4 when determining start and end positions of GI, sometimes these GI predictions overlapped. In this situation, the overlapping GI were combined. The GI labeled on the BRIG map were merged in this fashion. The nucleotide sequence of each genomic island of NADC 6564 was also used as a query against the published chromosomal sequences of reference strains EDL933 and Sakai to determine the presence and location of homologous GI in the reference strains. Exact pairs of repeats and exact tandem repeats were discovered in the chromosome of NADC 6564 by utilizing Mummer 3.23 [93]. Repeat-match program of mummer package identifies maximal exact repeats within a single sequence. This program simply exploits Mummer’s ability to align two different sequences based on sequence homology by applying it to one single sequence. The minimum match length option was set to 50 nucleotides.

### Whole chromosomal sequence alignments

In order to construct and visualize whole chromosomal alignments, a multiple genome alignment tool called Mauve was used [94]. Mauve compares multiple genome sequences and finds regions of homology called locally collinear blocks (LCB). The progressive Mauve algorithm was used with the default parameters in order to generate an alignment file in XMFA format. This XMFA file was then visualized using the Mauve GUI. Upon inspection of the initial Mauve alignment, the beginning of the chromosomal sequence of NADC 6564 [28] contained 6 LCB that were found at the end of the genomes in the other strains. In order to rearrange the LCB so that all of the sequences are uniform, the XMFA file was manually parsed to find the exact location of the first six LCB. The chromosomal sequence of strain 6564 was then modified so that the nucleotide sequence of the first six LCB were moved to the end.

### Evolutionary trees

#### Core genome comparison

In order to create a meaningful phylogenetic tree comparing NADC 6564 to other *E. coli* strains, the core genomes of 40 different strains were compared. To accomplish this, a FASTA file containing a list of genes and their nucleotide sequences was downloaded from the NCBI database for each genome compared (Additional file 6: Table S1). Next, all of the FASTA files downloaded were then concatenated into one FASTA file containing a list of all of the genes and respective nucleotide sequences from all of the strains being compared. This FASTA file was used as input for the program CD-HIT in order to cluster these genes into groups of equal length with similarity of 90% and above [95, 96]. The resulting clusters were filtered so that only the clusters containing exactly one gene from each chromosomal genome remained. This ensures that the gene is a core gene present in all of the strains compared. A total of 2284 core genes were identified among the 40 strains being compared. Once the clusters containing core genes were isolated, the clusters were split so that a FASTA file could be created for each strain containing core genes from the respective strain. These files are not identical because CD-HIT isolated the genes that are 90% homologous. Although the FASTA files aren’t identical, all of the FASTA files contain the same set of genes with the nucleotide sequence allowed to vary by 10% at most when compared across genomes. These core genomes were combined into a single multiFASTA file and used to infer a maximum likelihood phylogenetic tree using IQ-TREE [97]. The IQ-TREE phylogenetic inference consisted of using the model finder option (−m TESTNEW) to find the best evolutionary model appropriate for the input data [98]. Once the GTR + R4 was identified as the best evolutionary model, a maximum likelihood tree was constructed using IQ-TREE. Support values drawn by IQ-TREE were found using 1000 bootstrap replicates in combination with both the ultrafast bootstrap approximation method as well as the SH-like approximate ratio test method [99, 100]. The generated tree was visualized using FigTree using the midpoint rooting method [101]. Since there was no outgroup included in the tree, FigTree’s midpoint rooting method was used to root the tree.

### ParSNP-Gingr

Core phylogeny was also inferred using the parSNP-Gingr pipeline [102]. Whole genome sequences being compared were provided as input to parSNP using the NADC 6564 annotated GenBank file as the reference. Trees were created by visualizing the parSNP output in Gingr [102].

### Determination of DNA backbone S-modification (phosphorothioation) activity

The DNA backbone S-modification was assessed by treating genomic DNA of strains carrying (NADC 6564 and NADC 6565) or lacking (EDL933 and Sakai) *dndBCDE* operon, which encode enzymes for this activity [57], with 30 mM iodine solution for 15 min at 60 °C. The treated DNA was cooled to 4 °C and analyzed on a 1% agarose gel by a standard agarose gel electrophoresis procedure [57]. The strain NADC 6565 is a Congo red-positive variant of NADC 6564 and it also carries the *dndBCDE* operon based on the published genome sequence of these two strains [28]. Therefore, strain NADC 6565 was used as a positive control for confirming the presence of S-DNA modification activity in other strains tested in this assay.

### Determination of *dpt* encoded restriction system activity against DNA lacking S-modification

A high copy plasmid (pUC19) lacking the S-DNA modification was transformed by electroporation into *E. coli* strains harboring or lacking both *dndBCDE* and *dptFGH* operons, which encode DNA S-modification and a restriction system for restricting DNA lacking the S-modification, respectively. Electroporations were performed using a Gene Pulser (Bio-Rad Laboratories, Inc., Hercules, CA) and according to the manufacturer’s instructions. The transformed *E. coli* cells were diluted 1:10 in SOC (Super Optimal broth with Catabolite repression) broth (Bio 101, Inc., La Jolla, CA) and incubated for one hour at 37 °C on a shaker set to 200 rpm. Ten-fold serial dilutions of these cell suspensions were spread-plated on LB agar containing carbenicillin (100 μg/mL) and plates were incubated overnight at 37 °C. The number of colonies produced on the overnight-incubated plates from three independent experiments were counted and plotted.

### Determination of minimum inhibitory concentration (MIC) of colistin

Bacterial strains were grown overnight in LB broth (strains Sakai, EDL33, and TOP10), LB broth containing 100 μg/mL streptomycin (strain NADC 6564), or LB broth containing 24 μg/mL of colistin (*E. coli* strain BEAR 119605; kindly provided by Dr. Heather Allen, Food Safety and Enteric Pathogens Research Unit, National Animal Disease Center, ARS-USDA, Ames, Iowa, USA) at 37 °C on a shaker (200 rpm). The overnight cultures were diluted to 5 × 10^6^ cells/mL in Mueller Hinton Broth (MHB) containing 12.5 mg/L magnesium chloride and 12.5 mg/L calcium chloride (MHB-MC). Aliquots (100 μl) of diluted culture were added to the wells of a 96-well plate containing 100 μl of MHB-MC or MHB-MC supplemented with colistin (starting at 0.1 μg/mL in the first well and increasing by an order of 2-fold in succeeding wells so that the last well of the row contained 64 μg/mL of colistin). The plates were incubated at 37 °C for 18 to 24 h and wells were examined for any visible bacterial growth. The wells with the lowest concentration showing no visible bacterial growth were accepted as the MIC of colistin for that particular bacterial strain.

### Determination of evolutionary relationship of genes encoding phosphoethanolamine transferases and proteins mediating S-DNA modification (Dnd proteins) and restriction of unmodified DNA (Dpt proteins)

The phylogenetic trees displaying the evolutionary similarity of phosphoethanolamine transferases, Dnd, and Dpt proteins encoded in NADC 6564 were generated using IQ-TREE [97]. To construct an evolutionary tree of phsophoethanolamine transferases, the amino acid sequences for the phosphoethanolamine transferases of strains NADC 6564, Sakai, EDL933, FRIK2533, *E. coli* O111:NM, and *E. coli* O26:H11 were downloaded from the NCBI database. In addition, amino acid sequences of *mcr* genes encoding phosphoethanolamine transferases mediating mobilizable colistin resistance in different bacterial species were included in this comparison [103]. Both the Dnd and Dpt trees were generated by using alignments of these protein sequences that were acquired from the database at NCBI [90, 91]. The amino acid sequences for respective proteins were aligned using the MUSCLE alignment tool with default parameters [104]. The alignment files for each respective protein search was used as input for IQ-TREE [97]. IQ-TREE phylogenetic inference consisted of using the -m TESTNEW option in order to identify the best-fit evolutionary model according to the Bayesian Information Criterion [98]. Once GTR + I + G4 was identified as the best evolutionary model for both Dpt and Dnd data, a maximum likelihood tree was generated using IQ-TREE. Support values drawn by IQ-TREE were found using 1000 bootstrap replicates in combination with both the ultrafast bootstrap approximation method as well as the SH-like approximate ratio test method [99, 100]. All phylogenetic trees were visualized in FigTree using the midpoint rooting method if no outgroup was provided [101].

### Statistical analyses

A two sample, Student’s t-test was used to determine the significance of the difference in the number of carbenicilln-resistant colonies recovered from NADC 6564 and EDL933 after transformation with pUC19. Statistical significance of the difference in the minimum inhibitory concentration of colistin between NADC 6564 and the reference strains was assessed using one-way analysis of variance with multiple comparison of means. Data were analyzed with GraphPad Prism7 software (GraphPad Software, La Jolla, CA). The difference was considered significant at *p* < 0.05.

### Disclaimer

Mention of trade names or commercial products in this article is solely for the purpose of providing specific information and does not imply recommendation or endorsement by the U.S. Department of Agriculture. USDA is an equal opportunity provider and employer.

## Additional files

Additional file 1: **Table S2.** Sequence length and location of chromosomal regions in two reference strains exhibiting homology to bacteriophage regions of NADC 6564. (DOCX 33 kb)

Additional file 2: **Table S3.** Chromosomal location of genomic islands (GI) in NADC 6564 and corresponding homologs in two reference strains. (DOCX 38 kb)

Additional file 3: **Figure S1.** Cladograms showing evolutionary relationship of the *dndBCDE* gene sequences in NADC 6564 to other bacterial species. The nucleotide sequences of *dndBCDE* genes was used as a query (indicated in the red font) to download homologs of these genes. These homologous sequences were then used for constructing a maximum likelihood phylogenetic tree using IQ-TREE. The generated tree was visualized using FigTree. (DOCX 434 kb)

Additional file 4: **Figure S2.** Cladograms showing evolutionary relationship of the *dptFGH* gene sequences in NADC 6564 to other bacterial species. The nucleotide sequences of *dptFGH* genes was used as a query (indicated in the red font) to download homologs of these genes. These homologous sequences were then used for inferring a maximum likelihood phylogenetic tree using IQ-TREE. The generated tree was visualized using FigTree. (DOCX 293 kb)

Additional file 5: **Figure S3.** Cladograms showing evolutionary relationship of *pea* and *peaX* gene sequences of NADC 6564 to other bacterial species. The nucleotide sequences of *pea* and *peaX* genes were used as a query (indicated in the red font) to download homologs of these genes. These homologous sequences were then used for constructing a maximum likelihood phylogenetic tree using IQ-TREE. The generated tree was visualized using FigTree. (DOCX 234 kb)

Additional file 6: **Table S1.** Bacterial strains with their corresponding accession numbers used in the study. (DOCX 29 kb)

## Abbreviations

BLAST: The Basic Local Alignment Search Tool
BRIG: Blast Ring Image Generator
EPEC: enteropathogenic *E. coli*
GI: Genomic Island
LCB: Linear Conserved Blocks
LEE: Locus of Enterocyte Effacement
MHB: Mueller-Hinton Broth
MIC: Minimum Inhibitory Concentration
PEA: Phosphoethanolamine transferase
STEC: Shiga toxin-producing *E. coli*

## References

[1] Ackers ML, Mahon BE, Leahy E, Goode B, Damrow T, Hayes PS, Bibb WF, Rice DH, Barrett TJ, Hutwagner L (1998). An outbreak of *Escherichia coli* O157:H7 infections associated with leaf lettuce consumption. J Infect Dis.

[2] Bell BP, Goldoft M, Griffin PM, Davis MA, Gordon DC, Tarr PI, Bartleson CA, Lewis JH, Barrett TJ, Wells JG (1994). A multistate outbreak of *Escherichia coli* O157:H7-associated bloody diarrhea and hemolytic uremic syndrome from hamburgers. The Washington experience JAMA.

[3] Hilborn ED, Mermin JH, Mshar PA, Hadler JL, Voetsch A, Wojtkunski C, Swartz M, Mshar R, Lambert-Fair MA, Farrar JA (1999). A multistate outbreak of *Escherichia coli* O157:H7 infections associated with consumption of mesclun lettuce. Arch Intern Med.

[4] Keene WE, Hedberg K, Herriott DE, Hancock DD, McKay RW, Barrett TJ, Fleming DW (1997). A prolonged outbreak of *Escherichia coli* O157:H7 infections caused by commercially distributed raw milk. J Infect Dis.

[5] Rangel JM, Sparling PH, Crowe C, Griffin PM, Swerdlow DL (2005). Epidemiology of *Escherichia coli* O157:H7 outbreaks, United States, 1982-2002. Emerg Infect Dis.

[6] Banatvala N, Griffin PM, Greene KD, Barrett TJ, Bibb WF, Green JH, Wells JG (2001). Hemolytic uremic syndrome study C. The United States National Prospective Hemolytic Uremic Syndrome Study: microbiologic, serologic, clinical, and epidemiologic findings. J Infect Dis.

[7] Siegler R, Oakes R (2005). Hemolytic uremic syndrome; pathogenesis, treatment, and outcome. Curr Opin Pediatr.

[8] Hancock D, Besser T, Lejeune J, Davis M, Rice D (2001). The control of VTEC in the animal reservoir. Int J Food Microbiol.

[9] CDC (2006). *Escherichia coli* O157:H7 infection in children associated with raw milk and raw colostrum from cows—California. MMWR.

[10] Elder RO, Keen JE, Siragusa GR, Barkocy-Gallagher GA, Koohmaraie M, Laegreid WW (2000). Correlation of enterohemorrhagic *Escherichia coli* O157 prevalence in feces, hides, and carcasses of beef cattle during processing. Proc Natl Acad Sci U S A.

[11] Gyles CL (2007). Shiga toxin-producing *Escherichia coli*: an overview. J Anim Sci.

[12] Riley LW, Remis RS, Helgerson SD, McGee HB, Wells JG, Davis BR, Hebert RJ, Olcott ES, Johnson LM, Hargrett NT (1983). Hemorrhagic colitis associated with a rare *Escherichia coli* serotype. N Engl J Med.

[13] System C-NOR (2018). Disease outbreak data for 1998–2016.

[14] Davis MA, Hancock DD, Besser TE, Call DR (2003). Evaluation of pulsed-field gel electrophoresis as a tool for determining the degree of genetic relatedness between strains of *Escherichia coli* O157:H7. J Clin Microbiol.

[15] Davis MA, Hancock DD, Besser TE, Rice DH, Hovde CJ, Digiacomo R, Samadpour M, Call DR (2003). Correlation between geographic distance and genetic similarity in an international collection of bovine faecal *Escherichia coli* O157:H7 isolates. Epidemiol Infect.

[16] Elhadidy MM, Elkhatib WF (2015). Multilocus genotypic characterization of *Escherichia coli* O157:H7 recovered from food sources. Epidemiol Infect.

[17] Noller AC, McEllistrem MC, Harrison LH (2004). Genotyping primers for fully automated multilocus variable-number tandem repeat analysis of *Escherichia coli* O157:H7. J Clin Microbiol.

[18] Mellor GE, Besser TE, Davis MA, Beavis B, Jung W, Smith HV, Jennison AV, Doyle CJ, Chandry PS, Gobius KS (2013). Multilocus genotype analysis of *Escherichia coli* O157 isolates from Australia and the United States provides evidence of geographic divergence. Appl Environ Microbiol.

[19] Bustamante AV, Lucchesi PM, Parma AE (2009). Molecular characterization of Verocytotoxigenic *Escherichia coli* O157:H7 isolates from Argentina by multiple-loci VNTR analysis (MLVA). Braz J Microbiol.

[20] Heuvelink AE, van de Kar NC, Meis JF, Monnens LA, Melchers WJ (1995). Characterization of verocytotoxin-producing *Escherichia coli* O157 isolates from patients with haemolytic uraemic syndrome in Western Europe. Epidemiol Infect.

[21] Kim J, Nietfeldt J, Benson AK (1999). Octamer-based genome scanning distinguishes a unique subpopulation of *Escherichia coli* O157:H7 strains in cattle. Proc Natl Acad Sci U S A.

[22] Ziebell K, Steele M, Zhang Y, Benson A, Taboada EN, Laing C, McEwen S, Ciebin B, Johnson R, Gannon V (2008). Genotypic characterization and prevalence of virulence factors among Canadian *Escherichia coli* O157:H7 strains. Appl Environ Microbiol.

[23] Jung WK, Bono JL, Clawson ML, Leopold SR, Shringi S, Besser TE (2013). Lineage and genogroup-defining single nucleotide polymorphisms of *Escherichia coli* O157:H7. Appl Environ Microbiol.

[24] Zhang W, Qi W, Albert TJ, Motiwala AS, Alland D, Hyytia-Trees EK, Ribot EM, Fields PI, Whittam TS, Swaminathan B (2006). Probing genomic diversity and evolution of *Escherichia coli* O157 by single nucleotide polymorphisms. Genome Res.

[25] Eppinger M, Mammel MK, Leclerc JE, Ravel J, Cebula TA (2011). Genomic anatomy of *Escherichia coli* O157:H7 outbreaks. Proc Natl Acad Sci U S A.

[26] Eppinger M, Mammel MK, Leclerc JE, Ravel J, Cebula TA (2011). Genome signatures of *Escherichia coli* O157:H7 isolates from the bovine host reservoir. Appl Environ Microbiol.

[27] Kulasekara BR, Jacobs M, Zhou Y, Wu Z, Sims E, Saenphimmachak C, Rohmer L, Ritchie JM, Radey M, McKevitt M (2009). Analysis of the genome of the *Escherichia coli* O157:H7 2006 spinach-associated outbreak isolate indicates candidate genes that may enhance virulence. Infect Immun.

[28] Sharma VK, Bayles DO, Alt DP, Looft T (2016). Complete Genome Sequences of Curli-Negative and Curli-Positive Isolates of Foodborne *Escherichia coli* O157:H7 Strain 86–24. Genome Announc.

[29] Yang Z, Kovar J, Kim J, Nietfeldt J, Smith DR, Moxley RA, Olson ME, Fey PD, Benson AK (2004). Identification of common subpopulations of non-sorbitol-fermenting, beta-glucuronidase-negative *Escherichia coli* O157:H7 from bovine production environments and human clinical samples. Appl Environ Microbiol.

[30] Manning SD, Motiwala AS, Springman AC, Qi W, Lacher DW, Ouellette LM, Mladonicky JM, Somsel P, Rudrik JT, Dietrich SE (2008). Variation in virulence among clades of *Escherichia coli* O157:H7 associated with disease outbreaks. Proc Natl Acad Sci U S A.

[31] Parker CT, Kyle JL, Huynh S, Carter MQ, Brandl MT, Mandrell RE (2012). Distinct transcriptional profiles and phenotypes exhibited by *Escherichia coli* O157:H7 isolates related to the 2006 spinach-associated outbreak. Appl Environ Microbiol.

[32] Carter MQ, Brandl MT, Louie JW, Kyle JL, Carychao DK, Cooley MB, Parker CT, Bates AH, Mandrell RE (2011). Distinct acid resistance and survival fitness displayed by Curli variants of enterohemorrhagic *Escherichia coli* O157:H7. Appl Environ Microbiol.

[33] Carter MQ, Parker CT, Louie JW, Huynh S, Fagerquist CK, Mandrell RE (2012). RcsB contributes to the distinct stress fitness among *Escherichia coli* O157:H7 curli variants of the 1993 hamburger-associated outbreak strains. Appl Environ Microbiol.

[34] Sharma VK, Bayles DO, Alt DP, Looft T, Brunelle BW, Stasko JA (2017). Disruption of *rcsB* by a duplicated sequence in a curli-producing *Escherichia coli* O157:H7 results in differential gene expression in relation to biofilm formation, stress responses and metabolism. BMC Microbiol.

[35] Uhlich GA, Chen CY, Cottrell BJ, Hofmann CS, Dudley EG, Strobaugh TP, Nguyen LH (2013). Phage insertion in *mlrA* and variations in *rpoS* limit curli expression and biofilm formation in *Escherichia coli* serotype O157: H7. Microbiology.

[36] Uhlich GA, Keen JE, Elder RO (2001). Mutations in the *csgD* promoter associated with variations in curli expression in certain strains of *Escherichia coli* O157:H7. Appl Environ Microbiol.

[37] Carter MQ, Louie JW, Feng D, Zhong W, Brandl MT (2016). Curli fimbriae are conditionally required in *Escherichia coli* O157:H7 for initial attachment and biofilm formation. Food Microbiol.

[38] Edrington TS, Farrow RL, Sperandio V, Hughes DT, Lawrence TE, Callaway TR, Anderson RC, Nisbet DJ (2009). Acyl-homoserine-lactone autoinducer in the gastrointestinal [corrected] tract of feedlot cattle and correlation to season, *E. coli* O157:H7 prevalence, and diet. Curr Microbiol.

[39] Macarisin D, Patel J, Bauchan G, Giron JA, Sharma VK (2012). Role of curli and cellulose expression in adherence of *Escherichia coli* O157:H7 to spinach leaves. Foodborne Pathog Dis.

[40] Macarisin D, Patel J, Sharma VK (2014). Role of curli and plant cultivation conditions on *Escherichia coli* O157:H7 internalization into spinach grown on hydroponics and in soil. Int J Food Microbiol.

[41] Mahajan A, Currie CG, Mackie S, Tree J, McAteer S, McKendrick I, McNeilly TN, Roe A, La Ragione RM, Woodward MJ et al. An investigation of the expression and adhesin function of H7 flagella in the interaction of *Escherichia coli* O157 : H7 with bovine intestinal epithelium. Cell Microbiol 2009, 11(1):121–137. 10.1111/j.1462-5822.2008.01244.x 19016776

[42] Uhlich GA, Chen CY, Cottrell BJ, Andreozzi E, Irwin PL, Nguyen LH (2017). Genome amplification and promoter mutation expand the range of *csgD*-dependent biofilm responses in an STEC population. Microbiology.

[43] Uhlich GA, Keen JE, Elder RO (2002). Variations in the *csgD* promoter of *Escherichia coli* O157:H7 associated with increased virulence in mice and increased invasion of HEp-2 cells. Infect Immun.

[44] Yang Z, Kim J, Zhang C, Zhang M, Nietfeldt J, Southward CM, Surette MG, Kachman SD, Benson AK (2009). Genomic instability in regions adjacent to a highly conserved *pch* prophage in *Escherichia coli* O157:H7 generates diversity in expression patterns of the LEE pathogenicity island. J Bacteriol.

[45] Zhang Y, Laing C, Steele M, Ziebell K, Johnson R, Benson AK, Taboada E, Gannon VP (2007). Genome evolution in major *Escherichia coli* O157:H7 lineages. BMC Genomics.

[46] Zhou Z, Li X, Liu B, Beutin L, Xu J, Ren Y, Feng L, Lan R, Reeves PR, Wang L (2010). Derivation of *Escherichia coli* O157:H7 from its O55:H7 precursor. PLoS One.

[47] Perna NT, Plunkett G, Burland V, Mau B, Glasner JD, Rose DJ, Mayhew GF, Evans PS, Gregor J, Kirkpatrick HA (2001). Genome sequence of enterohaemorrhagic *Escherichia coli* O157:H7. Nature.

[48] Perna NT, Mayhew GF, Posfai G, Elliott S, Donnenberg MS, Kaper JB, Blattner FR (1998). Molecular evolution of a pathogenicity island from enterohemorrhagic *Escherichia coli* O157:H7. Infect Immun.

[49] Melton-Celsa A, Mohawk K, Teel L, O'Brien A (2012). Pathogenesis of Shiga-toxin producing *Escherichia coli*. Curr Top Microbiol Immunol.

[50] Naylor SW, Roe AJ, Nart P, Spears K, Smith DG, Low JC, Gally DL (2005). *Escherichia coli* O157 : H7 forms attaching and effacing lesions at the terminal rectum of cattle and colonization requires the LEE4 operon. Microbiology.

[51] Helminen M, Wisakanto KL, Raisio M, Baer M (1998). Enterohemorrhagic *Escherichia coli*--infection and a hemolytic uremic syndrome. Duodecim.

[52] Feng P, Lampel KA, Karch H, Whittam TS (1998). Genotypic and phenotypic changes in the emergence of *Escherichia coli* O157:H7. J Infect Dis.

[53] Whittam TS, Wolfe ML, Wachsmuth IK, Orskov F, Orskov I, Wilson RA (1993). Clonal relationships among *Escherichia coli* strains that cause hemorrhagic colitis and infantile diarrhea. Infect Immun.

[54] Zhou X, Deng Z, Firmin JL, Hopwood DA, Kieser T (1988). Site-specific degradation of *Streptomyces lividans* DNA during electrophoresis in buffers contaminated with ferrous iron. Nucleic Acids Res.

[55] Zhou X, He X, Liang J, Li A, Xu T, Kieser T, Helmann JD, Deng Z (2005). A novel DNA modification by Sulphur. Mol Microbiol.

[56] Yang Y, Xu G, Liang J, He Y, Xiong L, Li H, Bartlett D, Deng Z, Wang Z, Xiao X (2017). DNA backbone sulfur-modification expands microbial growth range under multiple stresses by its anti-oxidation function. Sci Rep.

[57] Xu T, Yao F, Zhou X, Deng Z, You D (2010). A novel host-specific restriction system associated with DNA backbone S-modification in *Salmonella*. Nucleic Acids Res.

[58] Ho WS, Ou HY, Yeo CC, Thong KL (2015). The *dnd* operon for DNA phosphorothioation modification system in *Escherichia coli* is located in diverse genomic islands. BMC Genomics.

[59] He X, Ou HY, Yu Q, Zhou X, Wu J, Liang J, Zhang W, Rajakumar K, Deng Z (2007). Analysis of a genomic island housing genes for DNA S-modification system in *Streptomyces lividans* 66 and its counterparts in other distantly related bacteria. Mol Microbiol.

[60] Borowiak M, Fischer J, Hammerl JA, Hendriksen RS, Szabo I, Malorny B (2017). Identification of a novel transposon-associated phosphoethanolamine transferase gene, *mcr-5*, conferring colistin resistance in d-tartrate fermenting *Salmonella enterica* subsp. *enterica* serovar Paratyphi B. J Antimicrob Chemother.

[61] Liu YY, Wang Y, Walsh TR, Yi LX, Zhang R, Spencer J, Doi Y, Tian G, Dong B, Huang X (2016). Emergence of plasmid-mediated colistin resistance mechanism MCR-1 in animals and human beings in China: a microbiological and molecular biological study. Lancet Infect Dis.

[62] Kim SH, Jia W, Parreira VR, Bishop RE, Gyles CL (2006). Phosphoethanolamine substitution in the lipid A of *Escherichia coli* O157 : H7 and its association with PmrC. Microbiology.

[63] Dean-Nystrom EA, Bosworth BT, Moon HW, O'Brien AD (1998). *Escherichia coli* O157:H7 requires intimin for enteropathogenicity in calves. Infect Immun.

[64] Dean-Nystrom EA, Gansheroff LJ, Mills M, Moon HW, O'Brien AD (2002). Vaccination of pregnant dams with intimin(O157) protects suckling piglets from *Escherichia coli* O157:H7 infection. Infect Immun.

[65] Dean-Nystrom EA, Melton-Celsa AR, Pohlenz JF, Moon HW, O'Brien AD (2003). Comparative pathogenicity of *Escherichia coli* O157 and intimin-negative non-O157 Shiga toxin-producing *E. coli* strains in neonatal pigs. Infect Immun.

[66] Dean-Nystrom EA, Pohlenz JF, Moon HW, O'Brien AD (2000). *Escherichia coli* O157:H7 causes more-severe systemic disease in suckling piglets than in colostrum-deprived neonatal piglets. Infect Immun.

[67] Sharma VK, Bearson BL (2013). Hha controls *Escherichia coli* O157:H7 biofilm formation by differential regulation of global transcriptional regulators FlhDC and CsgD. Appl Environ Microbiol.

[68] Sharma VK, Casey TA (2014). *Escherichia coli* O157:H7 lacking the *qseBC*-encoded quorum-sensing system outcompetes the parental strain in colonization of cattle intestines. Appl Environ Microbiol.

[69] Sharma VK, Kudva IT, Bearson BL, Stasko JA (2016). Contributions of EspA filaments and Curli fimbriae in cellular adherence and biofilm formation of Enterohemorrhagic *Escherichia coli* O157:H7. PLoS One.

[70] Sheng H, Nguyen YN, Hovde CJ, Sperandio V (2013). SdiA aids enterohemorrhagic *Escherichia coli* carriage by cattle fed a forage or grain diet. Infect Immun.

[71] Sinclair JF, Dean-Nystrom EA, O'Brien AD (2006). The established intimin receptor Tir and the putative eucaryotic intimin receptors nucleolin and beta1 integrin localize at or near the site of enterohemorrhagic *Escherichia coli* O157:H7 adherence to enterocytes *in vivo*. Infect Immun.

[72] Ogura Y, Ooka T, Iguchi A, Toh H, Asadulghani M, Oshima K, Kodama T, Abe H, Nakayama K, Kurokawa K (2009). Comparative genomics reveal the mechanism of the parallel evolution of O157 and non-O157 enterohemorrhagic *Escherichia coli*. Proc Natl Acad Sci U S A.

[73] Arndt D, Grant JR, Marcu A, Sajed T, Pon A, Liang Y, Wishart DS (2016). PHASTER: a better, faster version of the PHAST phage search tool. Nucleic Acids Res.

[74] Zhou Y, Liang Y, Lynch KH, Dennis JJ, Wishart DS (2011). PHAST: a fast phage search tool. Nucleic Acids Res.

[75] Alikhan NF, Petty NK, Ben Zakour NL, Beatson SA (2011). BLAST ring image generator (BRIG): simple prokaryote genome comparisons. BMC Genomics.

[76] Carter MQ, Louie JW, Huynh S, Parker CT (2014). Natural *rpoS* mutations contribute to population heterogeneity in *Escherichia coli* O157:H7 strains linked to the 2006 US spinach-associated outbreak. Food Microbiol.

[77] Liu C, Zheng H, Yang M, Xu Z, Wang X, Wei L, Tang B, Liu F, Zhang Y, Ding Y (2015). Genome analysis and *in vivo* virulence of porcine extraintestinal pathogenic *Escherichia coli* strain PCN033. BMC Genomics.

[78] An X, Xiong W, Yang Y, Li F, Zhou X, Wang Z, Deng Z, Liang J (2012). A novel target of IscS in *Escherichia coli*: participating in DNA phosphorothioation. PLoS One.

[79] Ou HY, He X, Shao Y, Tai C, Rajakumar K, Deng Z (2009). *dndDB*: a database focused on phosphorothioation of the DNA backbone. PLoS One.

[80] Gonzalez-Escalona N, MA MF, Rump LV, Payne J, Andrzejewski D, Brown EW, Evans PS, Croley TR. Draft Genome Sequences of Two O104:H21 *Escherichia coli* Isolates Causing Hemorrhagic Colitis during a 1994 Montana outbreak provide insight into their pathogenicity. Genome Announc. 2013, 1(5):e00805–13. 10.1128/genomeA.00805-13 PMC3790099 24092795

[81] Majowicz SE, Scallan E, Jones-Bitton A, Sargeant JM, Stapleton J, Angulo FJ, Yeung DH, Kirk MD (2014). Global incidence of human Shiga toxin-producing *Escherichia coli* infections and deaths: a systematic review and knowledge synthesis. Foodborne Pathog Dis.

[82] Ohnishi M, Kurokawa K, Hayashi T (2001). Diversification of *Escherichia coli* genomes: are bacteriophages the major contributors?. Trends Microbiol.

[83] Hayashi T, Makino K, Ohnishi M, Kurokawa K, Ishii K, Yokoyama K, Han CG, Ohtsubo E, Nakayama K, Murata T (2001). Complete genome sequence of enterohemorrhagic *Escherichia coli* O157:H7 and genomic comparison with a laboratory strain K-12. DNA Res.

[84] Laing CR, Buchanan C, Taboada EN, Zhang Y, Karmali MA, Thomas JE, Gannon VP (2009). In silico genomic analyses reveal three distinct lineages of *Escherichia coli* O157:H7, one of which is associated with hyper-virulence. BMC Genomics.

[85] Shaikh N, Tarr PI (2003). *Escherichia coli* O157:H7 Shiga toxin-encoding bacteriophages: integrations, excisions, truncations, and evolutionary implications. J Bacteriol.

[86] Shringi S, Schmidt C, Katherine K, Brayton KA, Hancock DD, Besser TE (2012). Carriage of *stx2a* differentiates clinical and bovine-biased strains of *Escherichia coli* O157. PLoS One.

[87] Wang G, Zhou F, Olman V, Li F, Xu Y (2010). Prediction of pathogenicity islands in enterohemorrhagic *Escherichia coli* O157:H7 using genomic barcodes. FEBS Lett.

[88] Brzuszkiewicz E, Thurmer A, Schuldes J, Leimbach A, Liesegang H, Meyer FD, Boelter J, Petersen H, Gottschalk G, Daniel R (2011). Genome sequence analyses of two isolates from the recent *Escherichia coli* outbreak in Germany reveal the emergence of a new pathotype: Entero-aggregative-Haemorrhagic *Escherichia coli* (EAHEC). Arch Microbiol.

[89] Wang L, Chen S, Vergin KL, Giovannoni SJ, Chan SW, DeMott MS, Taghizadeh K, Cordero OX, Cutler M, Timberlake S (2011). DNA phosphorothioation is widespread and quantized in bacterial genomes. Proc Natl Acad Sci U S A.

[90] Morgulis A, Coulouris G, Raytselis Y, Madden TL, Agarwala R, Schaffer AA (2008). Database indexing for production MegaBLAST searches. Bioinformatics.

[91] Zhang Z, Schwartz S, Wagner L, Miller W (2000). A greedy algorithm for aligning DNA sequences. J Comput Biol.

[92] Bertelli C, Laird MR, Williams KP (2017). Simon Fraser University research computing G, Lau BY, Hoad G, Winsor GL, brinkman FSL. IslandViewer 4: expanded prediction of genomic islands for larger-scale datasets. Nucleic Acids Res.

[93] Kurtz S, Phillippy A, Delcher AL, Smoot M, Shumway M, Antonescu C, Salzberg SL (2004). Versatile and open software for comparing large genomes. Genome Biol.

[94] Darling AE, Mau B, Perna NT (2010). progressiveMauve: multiple genome alignment with gene gain, loss and rearrangement. PLoS One.

[95] Fu L, Niu B, Zhu Z, Wu S, Li W (2012). CD-HIT: accelerated for clustering the next-generation sequencing data. Bioinformatics.

[96] Li W, Godzik A (2006). Cd-hit: a fast program for clustering and comparing large sets of protein or nucleotide sequences. Bioinformatics.

[97] Nguyen LT, Schmidt HA, von Haeseler A, Minh BQ (2015). IQ-TREE: a fast and effective stochastic algorithm for estimating maximum-likelihood phylogenies. Mol Biol Evol.

[98] Kalyaanamoorthy S, Minh BQ, Wong TKF, von Haeseler A, Jermiin LS (2017). ModelFinder: fast model selection for accurate phylogenetic estimates. Nat Methods.

[99] Guindon S, Dufayard JF, Lefort V, Anisimova M, Hordijk W, Gascuel O (2010). New algorithms and methods to estimate maximum-likelihood phylogenies: assessing the performance of PhyML 3.0. Syst Biol.

[100] Hoang DT, Chernomor O, von Haeseler A, Minh BQ, Vinh LS (2018). UFBoot2: improving the ultrafast bootstrap approximation. Mol Biol Evol.

[101] FigTree: http://tree.bio.ed.ac.uk/software/figtree/.

[102] Treangen TJ, Ondov BD, Koren S, Phillippy AM (2014). The harvest suite for rapid core-genome alignment and visualization of thousands of intraspecific microbial genomes. Genome Biol.

[103] Yin W, Li H, Shen Y, Liu Z, Wang S, Shen Z, Zhang R, Walsh TR, Shen J, Wang Y (2017). Novel Plasmid-Mediated Colistin Resistance Gene *mcr-3* in *Escherichia coli*. MBio.

[104] Edgar RC (2004). MUSCLE: multiple sequence alignment with high accuracy and high throughput. Nucleic Acids Res.

