# Supplementary material for: Comparative genomics reveals structural and functional features specific to the genome of a foodborne Escherichia coli O157:H7
Source: BMC Genomics. 2019 Mar 8;20:196. doi: 10.1186/s12864-019-5568-6 (PMC6408774; doi:10.1186/s12864-019-5568-6)
Supplement: Supplementary file 1 — Table S2. Sequence length and location of chromosomal regions in two reference strains exhibiting homology to bacteriophage regions of NADC 6564. (DOCX 33 kb) [file 12864_2019_5568_MOESM1_ESM.docx]

**Table S2** Sequence length and location of chromosomal regions in two reference strains exhibiting homology to bacteriophage regions of NADC 6564.

| Phage Regions of  NADC 6564/Phage  Names | Homologous Phage Regions Identified in^1^ | | | |  |  |
| --- | --- | --- | --- | --- | --- | --- |
|  | EDL933 Sakai | | | | |  |
|  | Sequence  length (kb) | Chromosomal  Location | Sequence  length (kb) | Chromosomal  location | Located in  Genomic  Island (GI) |  |
| 1/Stx2-converting  phage 1717 | 10.99 | 3568575-3579565 | 14.30 | 3477774-3492071 | GI-5 |  |
| 2/*Shigella* phage  Sf6 | 6.43 | 3278905-3285338 | 6.43 | 3192035-3198467 | GI-7 |  |
| 3/Enterobacteria  phage YYZ-2008 | 18.24 | 2982270-3000512 | 18.24 | 2894086-2912328 | GI-8 |  |
| 4/Enterobacteria  phage BP-4795 | 2.81 | 1535156-1537970 | 6.84 | 2742062-2748906 | GI-11 |  |
| 5/Enterobacteria  phage BP-4795 | 49.98 | 2771169-2821148 | 49.98 | 2676843-2726822 | GI-13 |  |
| 6/Enterobacteria  phage 933W | 30.35 | 1372476-1402824 | 21.21 | 1285952-1307157 | GI-15 |  |
| 7/Enterobacteria  phage BP-4795 | 34.68 | 1626386-1661044 | 53.18 | 1538786-1591970 | GI-20 |  |
| 8/*Brucella* phage  BiPBO1 | 15.23 | 1680282-1695510 | 15.23 | 1594844-1610072 | GI-21 |  |
| 9/*Escherichia* virus  Lambda | 24.21 | 1724306-1748520 | 27.79 | 1638868-1666662 | GI-23 |  |
| 10/Stx2-converting  phage 1717 | 2.64 | 1742143-1744779 | 2.64 | 1656705-1659341 | GI-23 |  |
| 11/Enterobacteria  phage YYZ-2008 | 113.55 | 1828825-1942373 | 55.10 | 1743776-1798875 | GI-25 |  |
| 12/Enterobacteria  phage BP-4795 | 60.09 | 2114833-2174923 | 33.89 | 1947201-1981089 | GI-30 |  |
| 13/Enterobacteria  phage BP-4795 | 57.42 | 2272753-2330172 | 41.73 | 2213290-2255023 | GI-32 |  |
| 14/Enterobacteria  phage P88 | 24.87 | 2686568-2711439 | 24.87 | 2592243-2617114 | GI-33 |  |
| 15/*Escherichia*  virus Lambda | 24.16 | 1265674-1289835 | 24.16 | 1178343-1202505 | GI-35 |  |
| 16/*Shigella* phage  POCJ13 | 24.18 | 1248629-1272806 | 24.18 | 1161298-1185475 | GI-35 |  |
| 17/Enterobacteria  phage cdtI | 48.19 | 890967-939158 | 48.19 | 891197-939388 | GI-37 |  |
| 18/Enterobacteria  phage SfI | 14.24 | 291223-305464 | 14.24 | 291222-305464 | GI-42 |  |
| 19/Acidianus tailed  spindle virus^2^ | 10.1 | Not present |  | Not present |  |  |

^1^ These chromosomal regions of EDL933 and Sakai strains exhibiting homology to phage regions of NADC 6564 were identified by BLAST using the individual phage regions of NADC 6564 as a query against published chromosomal sequences of EDL933 (Accession number: CP008957.1) and Sakai (Accession number: BA000007.3) strains.

^2^ Phage 19 is present only in NADC 6564 and it is located in GI-49 in the chromosome of this strain.
