# Supplementary material for: Comparative genomics reveals structural and functional features specific to the genome of a foodborne Escherichia coli O157:H7
Source: BMC Genomics. 2019 Mar 8;20:196. doi: 10.1186/s12864-019-5568-6 (PMC6408774; doi:10.1186/s12864-019-5568-6)
Supplement: Supplementary file 2 — Table S3. Chromosomal location of genomic islands (GI) in NADC 6564 and corresponding homologs in two reference strains. (DOCX 38 kb) [file 12864_2019_5568_MOESM2_ESM.docx]

**Table S3** Chromosomal location of genomic islands (GI) in NADC 6564 and corresponding homologs in two reference strains.

| Chromosomal locations of homologous Genomic Islands (GI) in strain | | | | | | | | | |
| --- | --- | --- | --- | --- | --- | --- | --- | --- | --- |
|  | NADC 6564^1^ | | | EDL933^1^ | | | Sakai^1^ | | |
| GI # | Start^2^ | Stop^3^ | Length | Start^2^ | Stop^3^ | Length | Start^2^ | Stop^3^ | Length |
| GI_1 | 45660 | 87369 | 41710 | 4670433 | 4712143 | 41710 | 4582934 | 4624644 | 41710 |
| GI_2 | 121204 | 126377 | 5174 | 4631425 | 4636598 | 5173 | 4543926 | 4549099 | 5173 |
| GI_3 | 796581 | 815957 | 19377 | 3943153 | 3962529 | 19376 | 3855654 | 3875030 | 19376 |
| GI_4 | 934362 | 961547 | 27186 | 3797563 | 3824748 | 27185 | 3710064 | 3737249 | 27185 |
| GI_5 | 1151792 | 1197911 | 46120^*^ | 3579460 | 3607318 | 27858 | 3491924 | 3519824 | 27900 |
| GI_6 | 1448295 | 1462748 | 14454 | 3295780 | 3310233 | 14453 | 3208909 | 3223362 | 14453 |
| GI_7 | 1468301 | 1478379 | 10079 | 3280149 | 3290227 | 10078 | 3193278 | 3203356 | 10078 |
| GI_8 | 1731299 | 1753225 | 21927 | 2985086 | 3000512 | 15426 | 2896902 | 2912328 | 15426 |
| GI_9 | 1780152 | 1785990 | 5839 | 2951008 | 2956846 | 5838 | 2862824 | 2868662 | 5838 |
| GI_10 | 1861428 | 1871868 | 10441 | 2865142 | 2875582 | 10440 | 2776970 | 2787410 | 10440 |
| GI_11 | 1897265 | 1910550 | 13286 | 2832611 | 2837594 | 4983 | 2738285 | 2751573 | 13288 |
| GI_12 | 1917295 | 1922367 | 5073 | 2820794 | 2825866 | 5072 | 2726468 | 2731540 | 5072 |
| GI_13 | 1935682 | 1942814 | 7133 | 2800345 | 2807479 | 7134 | 2706018 | 2713152 | 7134 |
| GI_14 | 1957660 | 1980399 | 22740 | 1277158 | 1297361 | 20203 | 1189827 | 1210031 | 20204 |
| GI_15 | 2021254 | 2063556 | 42303 | 1346562 | 1372616 | 26054 | 1271813 | 1286092 | 14279 |
| GI_16 | 2066136 | 2078210 | 12075 | 1383071 | 1395145 | 12074 | 1296547 | 1307157 | 10610 |
| GI_17 | 2100283 | 2113282 | 13000 | 1417217 | 1430216 | 12999 | 1330932 | 1343931 | 12999 |
| GI_18 | 2120477 | 2129747 | 9271 | 1437411 | 1446681 | 9270 | 1351126 | 1360396 | 9270 |
| GI_19 | 2140123 | 2226046 | 85924 | 1056906 | 1141047 | 84141 | 1370772 | 1456696 | 85924 |
| GI_20 | 2312198 | 2358987 | 46790 | 1630454 | 1661044 | 30590 | 1542854 | 1589649 | 46795 |
| GI_21 | 2364055 | 2368422 | 4368 | 1680155 | 1684522 | 4367 | 1594717 | 1599084 | 4367 |
| GI_22 | 2370408 | 2379518 | 9111 | 1686508 | 1695618 | 9110 | 1601070 | 1610180 | 9110 |
| GI_23 | 2389139 | 2435585 | 46447 | 1724306 | 1748520 | 24214 | 1638868 | 1663851 | 24983 |
| GI_24 | 2532088 | 2537145 | 5058 | 1846720 | 1851777 | 5057 | 1761671 | 1766728 | 5057 |
| GI_25 | 2542277 | 2586748 | 44472 | 1856909 | 1901426 | 44517 | 1771860 | 1798875 | 27015 |
| GI_26 | 2597105 | 2622745 | 25641 | 1911783 | 1937436 | 25653 | 2158486 | 2184137 | 25651 |
| GI_27 | 2666031 | 2673029 | 6999 | 1980722 | 1987720 | 6998 | 2108202 | 2115200 | 6998 |
| GI_28 | 2675890 | 2680217 | 4328 | 1990581 | 1994908 | 4327 | 2101014 | 2105341 | 4327 |
| GI_29 | 2728579 | 2738389 | 9811 | 2043270 | 2053080 | 9810 | 2042842 | 2052652 | 9810 |
| GI_30 | 2808968 | 2834542 | 25575 | 2123658 | 2149244 | 25586 | 1947201 | 1972264 | 25063 |
| GI_31 | 2843205 | 2848887 | 5683 | 2157907 | 2163589 | 5682 | 1932943 | 1938625 | 5682 |
| GI_32 | 2964637 | 3006885 | 42249 | 2279340 | 2317922 | 38582 | 2213290 | 2237236 | 23946 |
| GI_33 | 3362913 | 3370640 | 7728 | 2687590 | 2695317 | 7727 | 2593265 | 2600992 | 7727 |
| GI_34 | 3402906 | 3407049 | 4144 | 2727584 | 2731727 | 4143 | 2633258 | 2637401 | 4143 |
| GI_35 | 3435698 | 3442058 | 6361 | 2760376 | 2766737 | 6361 | 2666050 | 2672411 | 6361 |
| GI_36 | 3442122 | 3483813 | 41692 | 1252501 | 1289835 | 37334 | 1165170 | 1202505 | 37335 |
| GI_37 | 3719690 | 3757796 | 38107 | 890967 | 929063 | 38096 | 891197 | 929293 | 38096 |
| GI_38 | 4202489 | 4209147 | 6659 | 439537 | 446195 | 6658 | 439538 | 446196 | 6658 |
| GI_39 | 4275463 | 4280502 | 5040 | 368183 | 373221 | 5038 | 368183 | 373222 | 5039 |
| GI_40 | 4285969 | 4290379 | 4411 | 358306 | 362716 | 4410 | 358306 | 362716 | 4410 |
| GI_41 | 4295304 | 4300685 | 5382 | 348000 | 353381 | 5381 | 348000 | 353381 | 5381 |
| GI_42 | 4330856 | 4348307 | 17452 | 306670 | 317829 | 11159 | 306670 | 317829 | 11159 |
| GI_43 | 4372363 | 4384534 | 12172 | 263864 | 276017 | 12153 | 263864 | 276017 | 12153 |
| GI_44 | 4404391 | 4410826 | 6436 | 237578 | 244013 | 6435 | 237578 | 244013 | 6435 |
| GI_45 | 4623651 | 4630076 | 6426 | 18326 | 24751 | 6425 | 18326 | 24751 | 6425 |
| GI_46 | 4707247 | 4711963 | 4717 | 5483763 | 5488479 | 4716 | 5435018 | 5439734 | 4716 |
| GI_47 | 4749059 | 4756049 | 6991 | 5439677 | 5446667 | 6990 | 5390920 | 5397910 | 6990 |
| GI_48 | 4756143 | 4787204 | 31062 | 5408522 | 5439583 | 31061 | 5359765 | 5390826 | 31061 |
| GI_49 | 4790017 | 4806071 | 16055 | 1137931 | 1140248 | 2317 | 2747371 | 2750721 | 3350 |
| GI_50 | 4811692 | 4815715 | 4024 | Not Present | | | Not Present | | |
| GI_51 | 4842976 | 4847786 | 4811 | 5376803 | 5381631 | 4828 | 5328064 | 5332874 | 4810 |
| GI_52 | 4997646 | 5004786 | 7141 | 5219803 | 5226943 | 7140 | 5171064 | 5178204 | 7140 |
| GI_53 | 5443345 | 5450663 | 7319 | 4773910 | 4781228 | 7318 | 4686411 | 4693729 | 7318 |

^1^Accession numbers of chromosomal sequences: NADC 6564: NZ_CP017251.1; EDL933: CP008957.1; and Sakai (Accession number: BA000007.3).

^2^ Starting or first base pair of the GI at its insertion site in the chromosome.

^3^ Ending or the last base pair of the GI at its insertion site in the chromosome.

^*^ Yellow highlight indicates that these genomic islands in EDL933 and Sakai were different in size than NADC 6564.
