# Supplementary figures and images for: Comparative genomics reveals structural and functional features specific to the genome of a foodborne Escherichia coli O157:H7
Source: BMC Genomics. 2019 Mar 8;20:196. doi: 10.1186/s12864-019-5568-6 (PMC6408774; doi:10.1186/s12864-019-5568-6)

***dndBCDE* Evolutionary TREE**


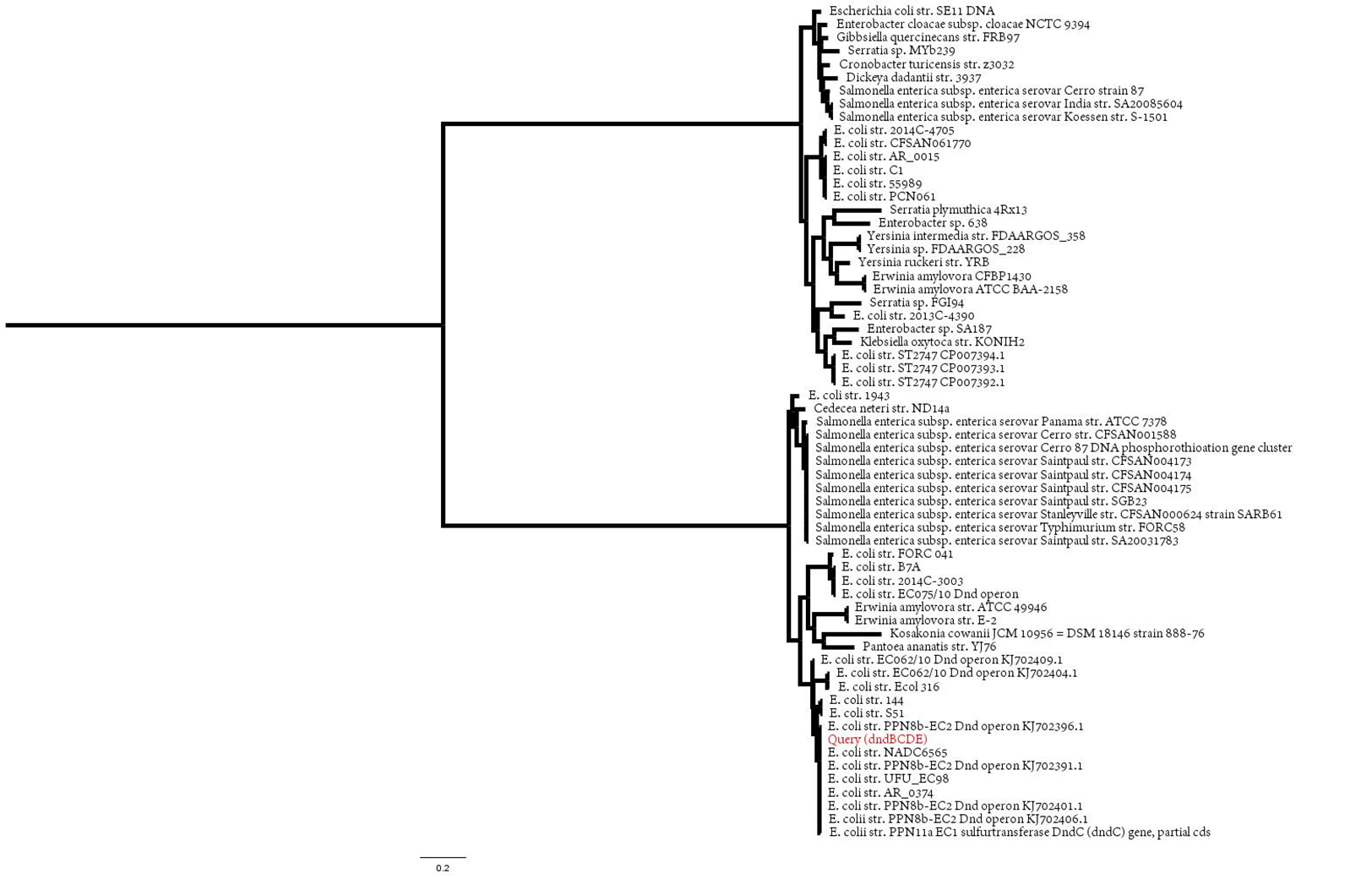

Supplement: Supplementary file 3 — Figure S1. Cladograms showing evolutionary relationship of the dndBCDE gene sequences in NADC 6564 to other bacterial species. The nucleotide sequences of dndBCDE genes was used as a query (indicated in the red font) to download homologs of these genes. These homologous sequences were then used for constructing a maximum likelihood phylogenetic tree using IQ-TREE. The generated tree was visualized using FigTree. (DOCX 434 kb) [file 12864_2019_5568_MOESM3_ESM.docx]

***dptFGH* Evolutionary TREE**


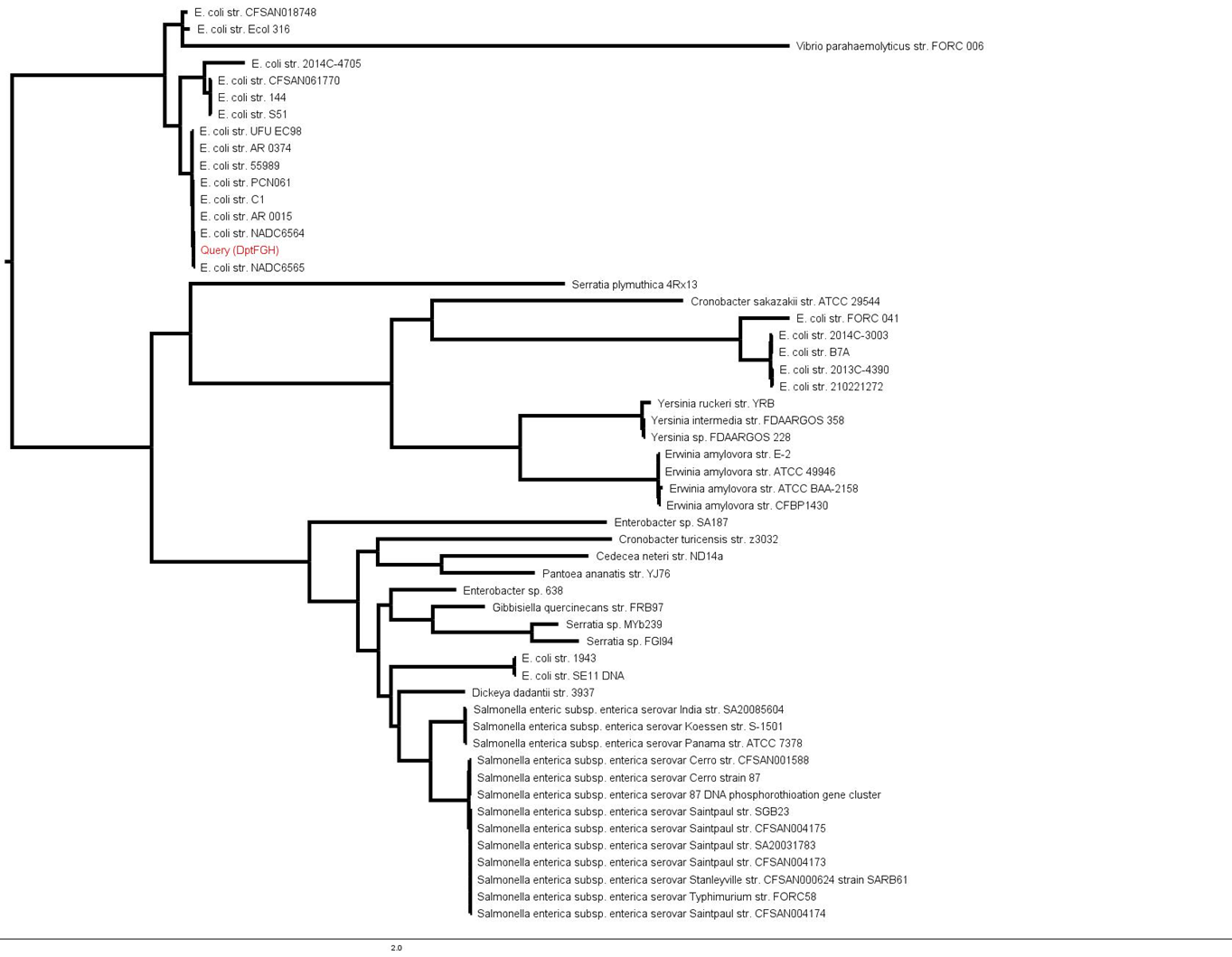

Supplement: Supplementary file 4 — Figure S2. Cladograms showing evolutionary relationship of the dptFGH gene sequences in NADC 6564 to other bacterial species. The nucleotide sequences of dptFGH genes was used as a query (indicated in the red font) to download homologs of these genes. These homologous sequences were then used for inferring a maximum likelihood phylogenetic tree using IQ-TREE. The generated tree was visualized using FigTree. (DOCX 293 kb) [file 12864_2019_5568_MOESM4_ESM.docx]

**PEAEvolutionary TREE**


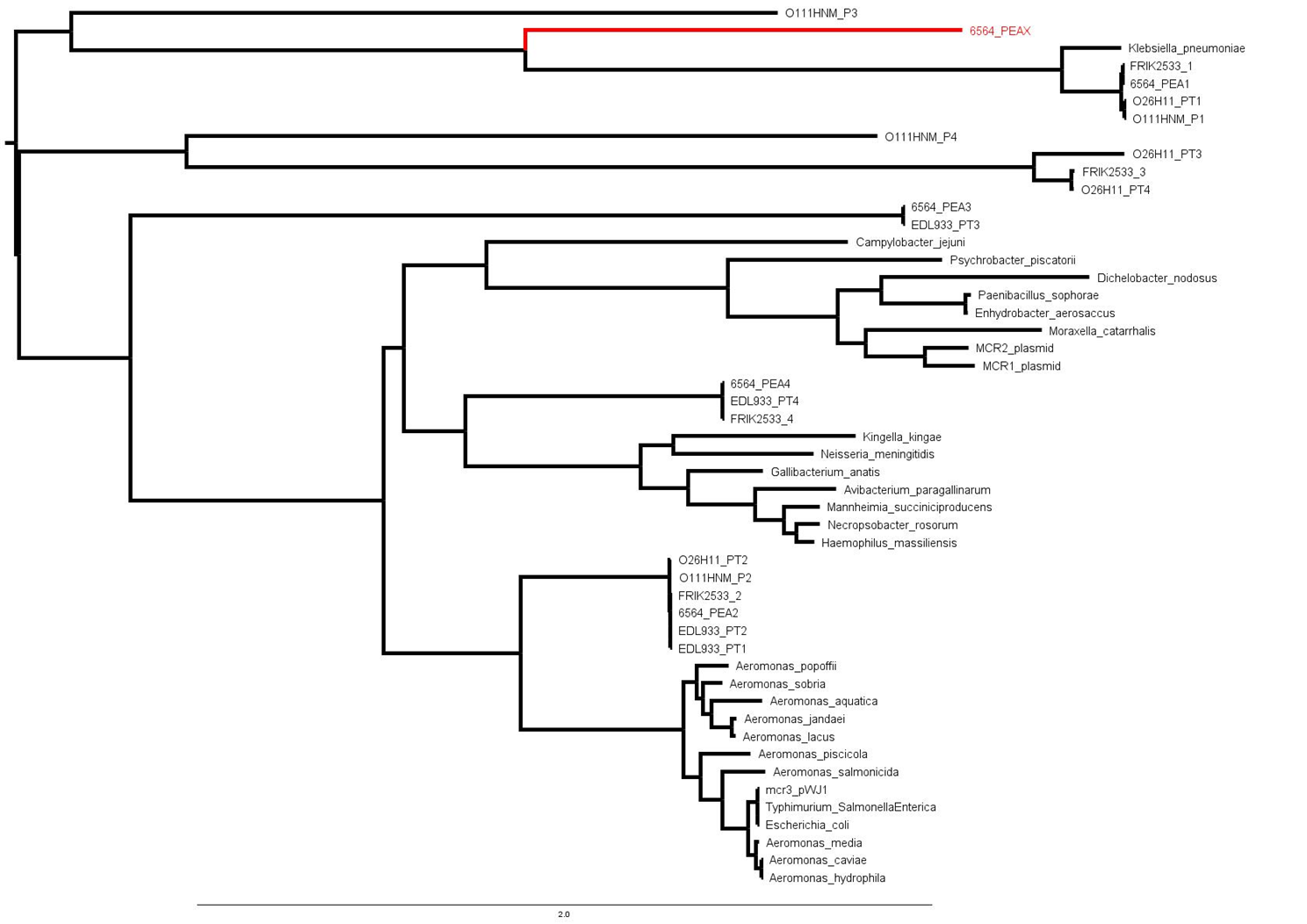

Supplement: Supplementary file 5 — Figure S3. Cladograms showing evolutionary relationship of pea and peaX gene sequences of NADC 6564 to other bacterial species. The nucleotide sequences of pea and peaX genes were used as a query (indicated in the red font) to download homologs of these genes. These homologous sequences were then used for constructing a maximum likelihood phylogenetic tree using IQ-TREE. The generated tree was visualized using FigTree. (DOCX 234 kb) [file 12864_2019_5568_MOESM5_ESM.docx]
