# Supplementary material for: Comparative genomics reveals structural and functional features specific to the genome of a foodborne Escherichia coli O157:H7
Source: BMC Genomics. 2019 Mar 8;20:196. doi: 10.1186/s12864-019-5568-6 (PMC6408774; doi:10.1186/s12864-019-5568-6)
Supplement: Supplementary file 6 — Table S1. Bacterial strains with their corresponding accession numbers used in the study. (DOCX 29 kb) [file 12864_2019_5568_MOESM6_ESM.docx]

**Additional Tables**

**Table S1** Bacterial strains with their corresponding accession numbers used in the study

| Bacterial Strains | Accession Number^1^ |
| --- | --- |
| *E. coli* O157:H7 str. FRIK2533 | NZ_CP015842.1B2:B35 |
| *E. coli* O157:H7 str. TW14359 | NC_013008.1 |
| *E. coli* O157:H7 str. Sakai | BA000007.3 |
| *E. coli* O18:K1:H7 str. UTI89 | NC_007946.1 |
| *E. coli* O157:H7 str. FRIK944 | NZ_CP016625.1 |
| *E. coli* O157:H7 str. Xuzhou21 | NC_017906.1 |
| *E. coli* O145:H28 str. RM12581 | NZ_CP007136.1 |
| *E. coli* O127:H6 str. E2348/69 | NC_011601.1 |
| *E. coli* O157:H7 NADC 6564 | NZ_CP017251.1 |
| *E. coli* K12 str. ER3413 | NZ_CP009789.1 |
| *E. coli* O157:H7 str. FRIK2455 | NZ_CP015843.2 |
| *E. coli* O157:H7 str. SS17 | NZ_CP008805.1 |
| *E. coli* O157:H7 str. SS52 | NZ_CP010304.1 |
| *E. coli* O157:H7 str. EC4115 | NC_011353.1 |
| *E. coli* O11 str. PCN033 | NZ_CP006632.1 |
| *E. coli* O139:H28 str. E24377A | NC_009801.1 |
| *E. coli* O55:H7 str. RM12579 | NC_017656.1 |
| *E. coli* O6:H1 str. CFT073 | NC_004431.1 |
| *E. coli* O145:H25 str. CFSAN004176 | NZ_CP014583.1 |
| *E. coli* O26:H11 str. 11368 | NC_013361.1 |
| *E. coli* O104:H4 str. 2011C-3493 | NC_018658.1 |
| *E. coli* O103:H2 str. 12009 | NC_013353.1 |
| *E. coli* O104:H4 str. 2009EL-2050 | NC_018650.1 |
| *E. coli* O157:H7 str. WS4202 | NZ_CP012802.1 |
| *E. coli* O145:H28 str. RM13514 | NZ_CP006027.1 |
| *E. coli* O104:H4 str. 2009EL-2071 | NC_018661.1 |
| *E. coli* O145:H28 str. RM13516 | NZ_CP006262.1 |
| *E. coli* O157:H7 str. 1130 | NZ_CP017434.1 |
| *E. coli* O78:H11 str. ETEC H10407 | NC_017633.1 |
| *E. coli* O157:H7 str. 8368 | NZ_CP017444.1 |
| *E. coli* O157:H7 str. 2149 | NZ_CP017436.1 |
| *E. coli* O2:K1:H5 str. APEC IMT5155 | NZ_CP005930.1 |
| *E. coli* K12 str. MG1655 | NC_000913.3 |
| *E. coli* O157:H7 str. EDL933 | NZ_CP008957.1 |
| *E. coli* O145:H28 str. RM12761 | NZ_CP007133.1 |
| *E. coli* O78 str. APEC O78 | NC_020163.1 |
| *E. coli* O6:K5:H1 str. Nissle 1917 | NZ_CP007799.1 |
| Salmonella Typhimurium LT2 | AE006468.2 |
| *E. coli* O104:H21 str. CFSAN002236 | NZ_CP023541.1 |
| *E. coli* O111:H- str. 11128 | NC_013364.1 |
| *E. coli* O1:K1:H7 str. APEC01 | NC_008563.1 |
| *E. coli* O157:H7 str. NADC 6565 | NZ_CP017249.1 |

^1^ These accession numbers were used to download annotated chromosomal sequences from the database at the NCBI.
